# Stable variable ranking and selection in regularized logistic regression for severely imbalanced big binary data

**DOI:** 10.1371/journal.pone.0280258

**Published:** 2023-01-17

**Authors:** Khurram Nadeem, Mehdi-Abderrahman Jabri

**Affiliations:** University of Guelph, Guelph, Ontario, Canada; University of Georgia, UNITED STATES

## Abstract

We develop a novel covariate ranking and selection algorithm for regularized ordinary logistic regression (OLR) models in the presence of severe class-imbalance in high dimensional datasets with correlated signal and noise covariates. Class-imbalance is resolved using response-based subsampling which we also employ to achieve stability in variable selection by creating an ensemble of regularized OLR models fitted to subsampled (and balanced) datasets. The regularization methods considered in our study include Lasso, adaptive Lasso (adaLasso) and ridge regression. Our methodology is versatile in the sense that it works effectively for regularization techniques involving both hard- (e.g. Lasso) and soft-shrinkage (e.g. ridge) of the regression coefficients. We assess selection performance by conducting a detailed simulation experiment involving varying moderate-to-severe class-imbalance ratios and highly correlated continuous and discrete signal and noise covariates. Simulation results show that our algorithm is robust against severe class-imbalance under the presence of highly correlated covariates, and consistently achieves stable and accurate variable selection with very low false discovery rate. We illustrate our methodology using a case study involving a severely imbalanced high-dimensional wildland fire occurrence dataset comprising 13 million instances. The case study and simulation results demonstrate that our framework provides a robust approach to variable selection in severely imbalanced big binary data.

## Introduction

Robust and efficient selection of covariates in logistic regression models is a core objective of statistical analysis in domains as diverse as epidemiology, forestry and insurance risk assessment as it enhances interpretation of fitted models and leads to improved prediction of the binary outcome (see, for example [1–3]). As compared to the ordinary logistic regression (OLR) model combined with traditional variable selection methods such as best subset selection, regularized versions of OLR have the potential to flag important variables in the presence of multicollinearity and are computationally attractive for high dimensional data. Bühlmann and Van De Geer [4] provide an excellent exposition of regularization techniques such as Lasso [5], elastic net [6], group Lasso [7], Dantzig selector [8] and SCAD [9].

Presence of rare events, as manifested through severe imbalance in observed frequencies of the binary classes, along with high dimensional and potentially multicollinear data can lead to serious challenges in deriving stable variable importance rankings in regularized regression models. For instance, it is well known that presence of a large number of irrelevant (noise) covariates having a strong correlation structure with the relevant (signal) covariates can seriously exacerbate variable selection in regularized regression models such as Lasso [10]. Class-imbalance occurs when one class is represented by a very small number of examples (minority class) as compared to the other (majority) class, where despite the fact that the majority class makes up most of the cases, it is the minority class that is often relevant for statistical analysis. Rarity of the minority class introduces problems and complications in conducting statistical inference [11, 12]. A severe minority-to-majority class ratio, such as in the range of 1:100 to 1:1000, often leads to very high volumes of data (big data) and significantly increases computational cost of model estimation [13].

The issue of severe class-imbalance can be addressed by employing response-based random sampling, which is done by sampling a subset of instances from both classes [14]. Response-based sampling is commonly used in case-control studies in epidemiological research and choice-based studies in econometrics [15–17]. Hosmer and Lemeshow [18] provide a thorough treatment of the effect of response-based sampling on estimation of the regression coefficients in logistic regression. A key advantage of response-based downsampling is that it results in substantially reduced datasets which in turn leads to a marked reduction in computational burden in training the models for data with millions of records. We refer the reader to [13, 19] for a detailed overview of sampling-based approaches in statistical and machine learning applications involving high class-imbalance in big binary data.

In this study, we employ response-based downsampling to develop a novel variable ranking and selection algorithm in the context of regularized OLR models. Our methodology consists of the following two key features: (i) repeated subsampling of the minority class (controls) to create a large number of balanced datasets (say *M)*, and (ii) computing stabilized aggregate covariate rank scores by generating an ensemble of regularized OLR model fits using the *M* balanced datasets created via case-control sampling. Our approach is similar in spirit to Bach’s [20] Bolasso algorithm which is based on replications of bootstrap sampling and intersecting the supports (i.e. covariates with nonzero coefficients) of the resulting Lasso bootstrap estimates. Bach [20] shows that this approach leads to consistent model selection whereas the original Lasso model estimated from a single dataset is shown to be inconsistent in the presence of strong correlations between signal and noise covariates [21]. Bolasso exploits the fact that bootstrap mimics availability of a large number of datasets by resampling from the same unique dataset and thereby diminishing the probability of selecting noise covariates from the intersected supports. We refer the read to [22–28] for examples of other recent approaches that involve the general notion of subsampling to perform variable selection in the context of machine learning based classifiers. However, recent variable selection literature, both in the context of machine learning classifiers and other regularized based methods, is focused on analyzing datasets that are moderate in size and with respect to class-imbalance severity. For example, highest sample size and the most severe class-imbalance ratio considered in [22–28] are 20,000 and 1:42 [26], respectively. In contrast, our methodology is designed to deal with variable selection in very large datasets in a computationally efficient manner, e.g. our case study models involve approximately 5 to 13 million observations with class-imbalance ratios around 1:500.

Here we replace the bootstrap sampling as employed in [20] with repeated response-based subsampling to create a large number of balanced datasets for variable ranking and selection in the presence of severe class-imbalance with potentially large volumes of data and varying degrees of multicollinearity. This study presents an extension to a heuristic variable ranking approach previously introduced in Nadeem et al. [29] who employed it to rank covariates in a large wildland fire occurrence dataset. Our work here is novel in that we rigorously develop the ranking approach, employ it to propose a new variable selection methodology for a general class of regularization methods, and thoroughly assess its selection performance by conducting an extensive simulation study. We demonstrate our framework using three regularization methods including Lasso [5, 30], adaLasso [21] and ridge regression [31]. Another important novel element of our stable variable ranking and selection (SVRS) methodology is its flexibility: it does not require hard shrinkage of the regression coefficients, as is the case with Lasso, and works equally well with the general class of regularization techniques that induce soft shrinkage only. We conduct a detailed simulation experiment and use a real-world case study involving big and highly imbalanced wildland fire occurrence datasets to show that, when compared to the usual practice where only a single regularized model fit is employed to determine variable importance, our variable selection approach successfully filters out the noise covariates and recovers a substantially higher proportion of signal covariates.

## Materials and methods

### The regularization methods

Binary response outcomes, observed as a negative (*y*_*i*_ = 0) or a positive (*y*_*i*_ = 01) occurrence from the *i*^*th*^ instance (example), can be modeled using the ordinary logistic regression model with the joint likelihood function for *n* instances given as: $L=\prod_{i=1}^{n}\pi_{i}^{y_{i}}(1-\pi_{i}^{y_{i}}{)}^{1-y_{i}}$, where *π*_*i*_ = *P*(*Y*_*i*_ = 1|***x***_*i*_) for a given vector of *p* covariate values, ***x***_***i***_. The probability of a positive occurrence is then parametrised via the logistic link function: $g\left(\pi_{i}\right)=\text{log}\left(\frac{\pi_{i}}{1-\pi_{i}}\right)=\alpha +x_{i}^{t}\beta$, where *α* is the intercept term and ***β*** is the vector of regression coefficients. The resulting negative log-likelihood function can be expressed as follows:

$$l\left(\beta \right)=\Sigma_{i=1}^{n}\left[\text{ln}\left(1+\text{exp}\left(\alpha +x_{i}^{t}\beta \right)\right)-y_{i}\left(\alpha +x_{i}^{t}\beta \right)\right].$$ (1)

#### Lasso

Lasso is a commonly employed regularization method for OLR [5, 30] where *l*_1_ penalty on *β* is imposed, leading to the following penalised form of (1):

$$l_{Lasso}\left(\beta \right)=\Sigma_{i=1}^{n}\left[\text{ln}\left(1+\text{exp}\left(\alpha +x_{i}^{t}\beta \right)\right)-y_{i}\left(\alpha +x_{i}^{t}\beta \right)\right]+\lambda \sum_{j=1}^{p}\left|\beta_{j}\right|\;,$$ (2)

where ${\left\|\beta \right\|}_{1}=\sum_{j=1}^{p}\left|\beta_{j}\right|$ is the *l*_1_-norm penalty, *λ* is a tuning parameter to be estimated separately and covariates typically enter in (2) in standardized form for the penalty to be meaningful. Lasso is a much more popular choice as compared to OLR for high dimensional data in that the *l*_1_ penalty allows simultaneous regularization of the coefficients and model selection by shrinking some of the coefficients to zero. Furthermore, efficient computational algorithms are available for computing the entire solution path for the tuning parameter λ for optimizing (2) [6, 30].

However, lasso does have its limitations including lack of robustness to high correlations among covariates and violation of the oracle property. A procedure is said to satisfy the oracle property if it estimates regression coefficients corresponding to noise covariates as zero with probability approaching to 1 and produces asymptotically unbiased and normally distributed estimates of the nonzero coefficients. Lasso can only perform consistent variable selection under strong conditions on the design matrix [21].

#### Adaptative lasso (adaLasso)

The adaLasso was introduced by Zou [21] which unlike the standard Lasso, is consistent in variable selection and satisfies the oracle property. Here the *l*_1_ penalty is modified by incorporating adaptive data-driven weights to the penalty. The adaLasso solution is obtained by maximizing the following penalized form of (1):

$$l_{AdaLasso}\left(\beta \right)=-\Sigma_{i=1}^{n}\left[\text{ln}\left(1+\text{exp}\left(\alpha +x_{i}^{t}\beta \right)\right)-y_{i}\left(\alpha +x_{i}^{t}\beta \right)\right]-\lambda \sum_{j=1}^{p}w_{j}\left|\beta_{j}\right|,$$ (3)

where wj's are the data-driven weights given as $w_{j}=1/{\left|{\hat{\beta }}_{j}^{*}\right|}^{\gamma };\;\gamma$ is a positive constant and ${\hat{\beta }}_{j}^{*}$ is an initial estimator of *β*_*j*_. Cross-validation can be employed to obtain optimal values of *γ* and *λ* from a grid of values where (0.5, 1, 2) are commonly used values of γ [21]. Here we use the standard Lasso solution for initial values ${\hat{\beta }}_{j}^{*}$ and set γ = 1 for simplicity of exposition.

#### Ridge regression

Ridge regression [31, 32] constraints the regression coefficients using the *l*_2_ norm penalty, ${\left\|\beta \right\|}_{2}=\sum_{j=1}^{p}\beta_{j}^{2}$, instead of the *l*_1_ penalty employed in (1). It tends to perform particularly well in the presence of strong multicollinearity among many covariates with potentially small effect sizes as it effectively regularizes the coefficients through a trade-off in bias and variance. As opposed to the Lasso solution, ridge regression produces even shrinkage for the correlated covariates [30]. A limitation of ridge regression is that it induces soft shrinkage, i.e. it does not force coefficients to vanish and therefore does not automatically perform variable selection, as is the case with Lasso and adaLasso.

The ridge regression based penalised log-likelihood function takes the following form:

$$l_{Ridge}\left(\beta \right)=\Sigma_{i=1}^{n}\left[\text{ln}\left(1+\text{exp}\left(\alpha +x_{i}^{t}\beta \right)\right)-y_{i}\left(\alpha +x_{i}^{t}\beta \right)\right]+\frac{\lambda }{2}\sum_{j=1}^{p}\beta_{j}^{2}.$$ (4)

The tuning parameter *λ* in (2–4) can be estimated using various methods including cross-validation [5], which we employ here in fitting models (2–4) using the R package *glmnet* [30].

### Class-imbalance and response-based sampling

As noted earlier, rarity of one of the binary response classes (usually the positive cases, Y = 1) can lead to significant imbalance in observed frequencies of the two classes. Datasets with severe class-imbalance are often prohibitively large and challenging for training statistical and machine learning models in a computationally tractable manner [33]. In this study we employ a response-based *downsampling* scheme for controls (*Y* = 0), where we retain all case instances and draw a simple random sample from controls. Let *n* be the size of the entire available sample and *n*_0_ and *n*_1_ (*n*_0_ > *n*_1_) be the total number of controls and cases, respectively; then *n*_1_ instances are randomly selected from the *n*_0_ controls resulting in a *balanced* (and therefore reduced) dataset of size *n*_b_ = 2*n*_1_. This has the effect of inducing an offset intercept term, log(*p*_1_/*p*_0_), into the *full-data* linear predictor $g\left(\pi_{i}\right)=\alpha +x_{i}^{t}\beta$, i.e. [18]:

$$g\left(\pi_{i}^{*}\right)=\text{log}\left(p_{1}/p_{0}\right)+\alpha +x_{i}^{t}\beta ,$$ (5)

where $\pi_{i}^{*}=Pr(Y_{i}=1|{\underline{x}}_{i},s_{i}=1)$, *s*_*i*_ is selection status (0 or 1) of the *i*^*th*^ instance in the original dataset; *p*_1_ and *p*_0_ are selection probabilities of cases and controls, respectively. These probabilities can be determined from respective sampling proportions. In case of the balanced sampling design employed in this study, *p*_1_ = 1 and *p*_0_ is estimated as the ratio of the number of controls to the number of cases in the full dataset.

### Stable Variable Ranking and Selection (SVRS) algorithm

Our variable ranking and selection algorithm is based on the following key steps:

1. Start with the full dataset of size *n*, *Z* = [***z***_1_, ***z***_2_, …, ***z***_*n*_], where $z_{i}={\left(y_{i},x_{i}^{t}\right)}^{t}$ is the *i*^th^ observed data vector and Z can be written as: Z = [*Z*_0_, *Z*_1_]; *Z*_0_ and *Z*_1_ denote data partitions corresponding to *y*_i_ = 0 (control) and *y*_i_ = 1 (case) observations, respectively.
2. For each *j* in (1, 2, …, *M*), do:
  - Draw a simple random sample of size *n*_1_, *Z*_0,*j*_, without replacement from control observations, *Z*_0_.
  - Generate the balanced dataset of size *n*_*b*_ as $Z_{j}^{\left(b\right)}=[Z_{0,j},Z_{1}]$.
  - Fit a regularized OLR model (e.g. Lasso) to $Z_{j}^{\left(b\right)}$ and store the estimated regression coefficients vector, ${\hat{\beta }}_{j}$.
3. Employ the variable ranking algorithm described in the next section to the resulting *M* × *p* coefficients matrix, $B={\left[{\hat{\beta }}_{1},\;{\hat{\beta }}_{2},\ldots ,{\hat{\beta }}_{M}\right]}^{t}$, to compute an aggregate rank score, *Rank*(*X*_*i*_), for each covariate *X*_*i*_, *i* = 1, 2, 3, …, *p*.
4. Choose a subset of variables as the most influential (relevant) covariates by selecting a threshold rank score from sorted rank scores, *Rank*(*X*_*i*_).

Next, we describe the variable ranking algorithm and our methodology for selecting a rank threshold for variable selection.

#### Ranking algorithm

For simplicity of exposition, we drop the *hat* symbol from the various quantities presented in this section, e.g. ${\hat{\beta }}_{j}$ is replaced by *β*_*j*_. Let us assume that the computed regression coefficients *β*_*j*_ are on their original scale of measurement (i.e., not standardized covariates) and let $\beta_{j}^{\Delta }={\left(\beta_{1,j}^{\Delta },\beta_{1,j}^{\Delta },\ldots ,\beta_{p,j}^{\Delta }\right)}^{t}$ denote the corresponding vector of *standardized coefficients* whose elements, $\beta_{i,j}^{\Delta }$, are defined as $\beta_{i,j}^{\Delta }=\text{sd}\left(x_{i}\right)\beta_{i,j}$, where sd(*x*_*i*_) is the standard deviation of the observed values for the *i*^*th*^ covariate. These standardized coefficients correspond to transformed covariates, $z_{k}=\frac{x_{k}-{\overset{-}{x}}_{k}}{{sd(x}_{k})}$.

Also, let (*R*_1,*j*_, *R*_2,*j*_, …, *R*_*p*,*j*_) be the ranks assigned to absolute values of $\beta_{i,j}^{\Delta }$ in the vector of standardized coefficients, namely $\left(\left|\beta_{1,j}^{\Delta }\right|,\left|\beta_{2,j}^{\Delta }\right|,\ldots ,\left|\beta_{p,j}^{\Delta }\right|\right)$, where 1 ≤ *R*_*i*,*j*_ ≤ *p*. Note that the covariates ranked at *P* and 1 have the highest and lowest values of the absolute standardized coefficients, respectively. After *sorting* in increasing order we denote resulting vector as ${\tilde{\beta }}_{j}=\left(\left|\beta_{i^{\left(1\right)},j}^{\Delta }\right|,\left|\beta_{i^{\left(2\right)},j}^{\Delta }\right|,\ldots ,\left|\beta_{i^{\left(p-1\right)},j}^{\Delta }\right|,\left|\beta_{i^{\left(p\right)},j}^{\Delta }\right|\right)$, such that $\left|\beta_{i^{\left(h\right)},j}^{\Delta }\right|\leq \left|\beta_{i^{\left(h+1\right)},j}^{\Delta }\right|$ and $X_{i^{\left(h\right)}}$ is the covariate whose assigned rank value is *h*. For instance, if *X*_5_ gets assigned a rank value of 10, we have *i*^(10)^ = 5. Note also that for a given rank position *h*, $X_{i^{\left(h\right)}}$ can differ between model fits for *j* = 1, 2, …, *M*.

An aggregate rank score based on *M* fits of the model is given by the following formula:

$$Rank\left(X_{i}\right)=M^{-1}\sum_{h=1}^{p}\sum_{j=1}^{M}hI_{R_{i,j}=h},$$ (6)

where *I*_*A*_ is an indicator function of event *A* and *h* indicates the rank positon.

*Theorem 1*. We have:

1. 1 ≤ *Rank*(*X*_*i*_) ≤ *p*, and
2. $\sum_{i=1}^{p}Rank\left(X_{i}\right)=\sum_{h=1}^{p}h=\frac{p\left(p+1\right)}{2}$.

Proof: i) Let us rewrite *Rank*(*X*_*i*_) as:

$$Rank\left(x_{i}\right)=M^{-1}\sum_{j=1}^{M}\sum_{h=1}^{p}hI_{R_{i,j}=h}.$$

Then we must have $1\leq \sum_{h=1}^{p}hI_{R_{i,j}=h}\leq p$ for a given *j*. So, the average over the index *j* must also satisfy the same constraints.

ii) Consider the sum

$$\begin{array}{ll} \sum_{i=1}^{P}Rank\left(x_{i}\right) & =M^{-1}\sum_{i=1}^{p}\sum_{j=1}^{M}\sum_{h=1}^{p}hI_{R_{i,j}=h},\\ & =M^{-1}\sum_{j=1}^{M}\sum_{h=1}^{p}h\left(\sum_{i=1}^{p}I_{R_{i,j}=h}\right),\\ & =M^{-1}\sum_{j=1}^{M}\sum_{h=1}^{p}h\left(1\right),\\ & =M^{-1}\sum_{j=1}^{M}\left(\sum_{h=1}^{p}h\right), \end{array}$$

$$\sum_{i=1}^{P}Rank\left(x_{i}\right)=\sum_{h=1}^{p}h=\frac{p\left(p+1\right)}{2}.$$

For regularization methods with hard shrinkage, such as Lasso logistic regression, it is common for several of the estimated coefficients to be forced to zero values at convergence as a consequence of the *l*_1_ regularization penalty. When displaying a ranking of estimated coefficients based on Lasso logistic fit, these *zeroed-out* coefficients need not be included in the ranking as they have no effect on predictive performance. A modified version of (1) is therefore presented as follows.

Let x* be a covariate having a zero coefficient in some *j*^th^ Lasso fit. Suppose x* is ranked at 10 in a particular fit, then its rank contribution in (1) from this fit is *h* = 10; even though its estimated coefficient is 0. However, we should penalize x* for this as it was in fact dropped from selection in that *j*^*th*^ fit. This is achieved by restructuring (1) as follows:

$$Rank\left(X_{i}\right)=M^{-1}\sum_{j=1}^{M}\sum_{h=1}^{p}hI_{R_{i,j}=h}I_{\left|\beta_{i,j}^{\Delta }\right|>0},$$ (7)

where *Rank*(*X*_*i*_) ≤ *p*, and $\sum_{i=1}^{p}Rank\left(X_{i}\right)\leq \sum_{h=1}^{p}h$.

A related scenario can also arise when several covariates are repeatedly zeroed-out in all *M* model fits, causing all the regression coefficients falling within a certain rank position to become zero. This leads us to the following definitions and a related theorem.

*Definition 1 (supported variables)*: The set of supported covariates is given as

$$\chi =\left\{x_{i}\in x:\Sigma_{j=1}^{M}\left|\beta_{i,j}^{\Delta }\right|>0\right\}.$$

Essentially, ***χ*** consists of all covariates that were not dropped in at least of the *M* model fits. For soft-shrinkage regularization such as in Ridge regression, we typically have ***χ*** = ***x***.

*Definition 2 (effective maximum rank)*: The effective maximum rank is given as:

$$p_{EF}=max\left\{h\in \left(1,\;2,\;\ldots ,p\right):\Sigma_{j=1}^{M}\Sigma_{i=1}^{p}\left|\beta_{i,j}^{\Delta }\right|I_{R_{i,j}=h}>0\right\},$$

where $\Sigma_{i=1}^{p}\left|\beta_{i,j}^{\Delta }\right|I_{R_{i,j}=h}=\left|\beta_{i^{\left(h\right)},j}^{\Delta }\right|$ for a given value of *h*, and *p*_*EF*_ ≤ *p*.

*Theorem 2*. Let $S_{h}=\Sigma_{j=1}^{M}\left|\beta_{i^{\left(h\right)},j}^{\Delta }\right|$, and assume that $S_{\tilde{h}}=0$ for some $\tilde{h}<p$, then we have *S*_*h*_ = 0 for all $h\geq \tilde{h}$.

Proof: $S_{\tilde{h}}=0$ implies that $\left|\beta_{i^{\left(\tilde{h}\right)},j}^{\Delta }\right|=0$ for all *j*. This also implies that $\left|\beta_{i^{\left(\tilde{h}+1\right)},j}^{\Delta }\right|$ must all be zero because, for any given *j*, we have by definition: $\left|\beta_{i^{\left(h\right)},j}^{\Delta }\right|\leq \left|\beta_{i^{\left(h+1\right)},j}^{\Delta }\right|$. Therefore, we must have $S_{\tilde{h}+1}=0$; and hence $S_{\tilde{h}+k}=0$ for all $1\leq k\leq (p-\tilde{h})$.

Therefore, considering Theorem 2, we further modify (7) as follows.

*Definition 3*. Let $\tilde{B}={\left[\left|{\tilde{\beta }}_{1}\right|,\;\left|{\tilde{\beta }}_{2}\right|,\ldots ,\left|{\tilde{\beta }}_{M}\right|\right]}^{t}$ be the *M* × *p* matrix whose entries (*j*, *h*) are given as $\left|\beta_{i^{\left(h\right)},j}^{\Delta }\right|$. A modified version of *Rank*(*X*_*i*_) for a maximum effective rank *p*_*EF*_, is defined as:

$$Rank\left(X_{i}\right)=M^{-1}\sum_{j=1}^{M}\sum_{h=1}^{p_{EF}}hI_{i=i^{\left(h\right)}}I_{\left|\beta_{i^{\left(h\right)},j}^{\Delta }\right|>0},$$ (8)

where *X*_*i*_ ∈ ***χ***, and the second summation on the right is taken over the first *p*_*EF*_ columns of $\tilde{B}$.

Here, (3) now satisfies the properties: i) 0 < *Rank*(*X*_*i*_) ≤ *p*_*EF*_ and, ii) $\sum_{i=1}^{p}Rank\left(X_{i}\right)\leq \sum_{h=1}^{p_{EF}}h=p_{EF}\left(p_{EF}+1\right)/2$, where *Rank*(*X*_*i*_) = 0 for *X*_*i*_ ∈ ***χ***^c^.

We use (8) as a basis for ranking covariates arising from the model fits. Note that (8) is equivalent to (6) when *χ* = *x*.

#### Threshold selection

Acquiring a list of covariates ranked by their relative importance obtained from the algorithm described above only completes the task of computing stable variable rank scores. Our objective here is to determine how many and which of these covariates are influential predictors in the logistic regression model. One simple approach to selecting important covariates is to examine selection metrics for choosing a threshold that partitions the covariates in influential and irrelevant sets. For instance, Nadeem et al. [29] used a selection metric, *p*_*drop*_, to determine the selection threshold. The metric *p*_*drop*_ is defined as: $p_{{Drop}_{i}}=\sum_{j=1}^{M}I_{\beta_{i,j}=0}/M$; that is, it denotes fraction of times the *i*^*th*^ covariate is dropped from Lasso logistic model fits. Nadeem et al. [29] then plotted $p_{{Drop}_{i}}$ values and noted that it tends to have a change point which they determined visually by choosing a threshold to separate the variables in clusters of important and irrelevant covariates (see Fig 8 in Nadeem et al. [29]). Notice that the notion of the *p*_*drop*_ metric used by Nadeem et al. [29] for variable selection is similar in spirit to the concept of empirical *selection probability* as introduced by Meinshausen and Bühlmann [34]. However, here we introduce *Rank*(*X*_*i*_) as the selection metric and show that it works well for both Lasso and other regularization methods that does not enforce hard shrinkage of the covariates.

We employ automatic thresholding of the rank scores based on change-point detection methodology [35–37]. Suppose the rank scores are ordered as a sequence of values (*r*_1_, *r*_2_, …, *r*_*v*_) and if there exists an index *τ* ∈ (1, 2, …, *v* − 1), such that some feature (e.g. mean) in the probability distribution of (*r*_1_, *r*_2_, …, *r*_*τ*_) and (*r*_*τ*+1_, *r*_*τ*+2_, …, *r*_*v*_) differ, then a change point has occurred. We employ the *changepoint* package [38] in R, to find a single change point in the mean *Rank*(*X*_*i*_) values. We refer the reader to Killick et al. [39] for further details on the detection tests implemented in the *changepoint* package.

#### Computational efficiency

Here we provide an illustration of computational efficiency of the SVRS algorithm in the context of the Lasso regularization method. Similar gains in efficiency are expected under other regularization techniques, such as ridge regression, due to the use of response-based subsampling. Computational complexity of Lasso algorithm is *O*(*np*^2^ + *p*^3^) [34] for *n* > *p* case considered in this study. The regularization parameter is selected in practice using *K*-fold cross-validation, which results in a computing time of *O*(*np*^2^ (*K* − 1) + *p*^3^). On the other hand, the cost for a single balanced dataset with *K*-fold cross-validation is *O*(2*n*_1_ (*K* − 1)*p*^2^ + *p*^3^), where *n*_1_ is the total number of cases in the entire dataset. Computational complexity for SVRS is therefore *O*(2*n*_1_*M*(*K* − 1)*p*^2^ + *p*^3^), when Lasso model is fitted to *M* balanced datasets. As we demonstrate in our case study analyses later, *M* = 100 is a reasonable choice for very large imbalanced datasets. Hence, algorithmic complexity of SVRS scales with the size of the minority class only. For instance, one of the datasets analyzed in our case study has *n* = 10.7 million and *n*_1_ = 22,525. Therefore, fitting the Lasso model to the *entire* original data would require approximately 238 times as many computational resources as needed to analyze a single balanced dataset. Whereas fitting the model to full data would still cost about 2.4 times more resources than running our SVRS algorithm.

It is however crucial to note that fitting regularized versions of the OLR model to massively large datasets is often computationally infeasible due to restricted amount of memory (RAM) available on computers. This issue is especially exacerbated for commonly used analysis languages such as R. The SVRS algorithm is therefore attractive in the sense that: i) it circumvents the computational bottleneck by making it feasible to estimate the model from much smaller subsets of the original data, and ii) its implementation is highly parallelizable which yields further gains in computational efficiency. For the abovementioned case study example, parallelized implementation of Lasso based SVRS algorithm (*M* = 100, *p* = 82) only took about 7.5 minutes on a 3.4-GHz machine with 16 cores, using *glmnet* R package [30].

### Simulation experiment

We simulate data under (1) using four OLR models with varying number of signal and noise covariates where two of them include correlated noise and signal covariates, while the remaining two models do not possess correlated covariates. Model descriptions along with respective ratios of number of signal to number of noise covariates, *s*: *m*; and regression coefficient vectors, are reported in Table 1.

**Table 1:** Number of covariates (*p*), ratio of number of signal to number of noise covariates (*s*: *m*), and the nonzero regression coefficients corresponding to the signal covariates for the four simulation models.

Simulation Model	*p* (*s*: *m*)	Correlated Covariates	Signal Coefficients (*β*_*i*_ ≠ 0)
UNCOR-12	100 (12:88)	No	(-0.9, -0.8, -0.7, -0.6, -0.5, -0.4, 1.0, 1.1, 1.2, 1.3, 1.4, 1.5)
COR-12		Yes	
UNCOR-24	200 (24:176)	No	(-1.20, -1.10, -1.00, -0.90, -0.80, -0.70, -0.60, -0.50, -0.40, -0.30, -0.20, -0.10, 0.05, 0.15, 0.25, 0.35, 0.45, 0.55, 0.65, 0.75, 0.85, 0.95, 1.05, 1.15)
COR-24		Yes	

#### Simulation of model with uncorrelated covariates

Data under the models with uncorrelated covariates, UNCOR-12 and UNCOR-24, are generated as follows where (*X*_1_, *X*_2_, …., *X*_*s*_) are the signal covariates (Table 1).

*UNCOR-12*. Signal covariates are distributed as *X*_*j*_ (*j* = 1, 2,.., 6)~ *Bernoulli*(0.5) and *X*_*j*_ (*j* = 7, 8,.., 12) ~ *N*(0,1); whereas the noise covariates are *X*_*j*_ (*j* = 13, 14,.., 18)~*Bernoulli*(0.5) and *X*_*j*_ (*j* = 19, 20,.., 100) ~ *Unif*(0,1).

*UNCOR-24*. We have *X*_*j*_ (*j* = 1, 2,.., 12)~ *Bernoulli*(0.5) and *X*_*j*_ (*j* = 13, 8,.., 24) ~ *N*(0,1) generated as the signal covariates. Noise covariates are simulated as *X*_*j*_ ~ *Unif*(0,1), *j* = 25, …, 200.

All covariates under UNCOR-12 and UNCOR-24 are distributed as independent random variables. We also apply logistic transformation to the normally distributed covariates so that their support is the unit interval, [0,1]. This was done to ensure that the covariates scales are relatively consistent, and the magnitudes of regression coefficients (Table 1) reflect relative variable importance in the simulated OLR model. Distribution and correlation structure of the covariates under COR-12 and COR-24 simulation models are described in the subsection ahead.

#### Simulation of models with correlated covariates

We include both categorical and continuous correlated covariates in the COR-12 and COR-24 simulation models. Touloumis [40] provide a computational framework to simulate correlated clusters of categorical variables using marginal baseline-category logit models [41]. They describe and implement the NORmal-To-Anything (NORTA) method introduced by Cario and Nelson [42] for simulating correlated binary and nominal responses under a desired marginal model specification. We employ R package *SimCorMultRes* [40] to simulate data and use the NORTA method to generate data for categorical responses which we incorporate in our simulation models as categorical covariates.

Data generation process under COR-12 and COR-24 models is described as follows where the first *s* covariates (*X*_1_, *X*_2_, …, *X*_*s*_) are signal and the rest are all noise (Table 1).

*COR-12*. We generate four clusters of a 4-category nominal variable *Z* where $Z_{c,1}^{t}=\left(X_{1},X_{2},X_{3},X_{*1}\right)$ denote the four dummy variables (i.e. *X*_1_ + *X*_2_ + *X*_3_ + X_*1_ = 1) corresponding to the categories of ***Z***_*c*,1_ in cluster 1. Similarly, we have $Z_{c,2}^{t}=\left(X_{4},X_{5},X_{6},X_{*2}\right)$ and $Z_{c,3}^{t}=\left(X_{13},X_{14},X_{15},X_{*3}\right)$ for cluster 2 and 3 respectively, where $Z_{c,3}^{t}$ contains noise covariates. Here, $Z_{c,i}^{t}$ are all correlated, and we do not use X_*i_ to avoid perfect multicollinearity among the four dummy variables (e.g. $X_{*1}=\sum_{i=}^{3}X_{i}$). We do not also incorporate $Z_{c,4}^{t}$ in the COR-12 simulations. We further generate a separate set of correlated binary covariates (*X*_16_, *X*_17_, *X*_18_) independently from the categorical covariates.

Rest of the covariates are continuous and independently generated as follows: (*X*_7_, *X*_8_, …, *X*_12_)~*MVN*(**0**, *σ****R***), where off-diagonal entries *σ*_*k*,*l*_ of the correlation matrix ***R*** are all 0.8; and the remaining noise covariates (*X*_19_, *X*_20_, …, *X*_100_) are iid *Unif* (0,1).

*COR-24*. Here, we retain the four correlated clusters of the 4-category variable *Z* with the revised labeling $Z_{c,3}^{t}=\left(X_{25},X_{26},X_{27},X_{*3}\right)$ and $Z_{c,4}^{t}=\left(X_{28},X_{29},X_{30},X_{*4}\right)$ for cluster 3 and 4, respectively. Labels for $Z_{c,1}^{t}$ and $Z_{c,2}^{t}$ remain unchanged. Independently of ***Z***_*c*,*i*_, we simulate (*X*_7_, …, *X*_12_) and (*X*_31_, …, *X*_36_) as correlated binary covariates. Continuous signal covariates are simulated as follows:

$$\left(X_{13},\ldots ,X_{24}\right)\sim MVN\left(0,\sigma R\right),$$

where *σ*_*k*,*l*_ = 0.8 for the off-diagonal entries of ***R***; and rest of the noise covariates (*X*_37_, *X*_38_, …, *X*_200_) are iid *Unif*(0,1).

For COR-12 and COR-24, all normally distributed covariates were mapped to [0,1] using the logistic transformation.

#### Generation of balanced datasets

We create balanced datasets using response-based sampling performed on initial datasets (of size *n*) that are generated under the four simulation models with three class-imbalance ratios (*I*_*R*_) and increasing sizes (*n*_*b*_) of the balanced samples. Combinations of various (*I*_*R*_, *n*_*b*_) values considered in our simulation experiment along with initial sample sizes (*n*) required to ensure various imbalance ratios are reported in Table 2. We induce rarity of the positive class by incorporating appropriately small values of the intercept, *α*, in the simulation models. We generate *M* = 500 balanced datasets under each of the resulting 60 combinations involving four models, three *I*_*R*_ values and five *n*_*b*_ values (Tables 1 and 2). Lasso, adaLasso and ridge regression are then fitted to each balanced dataset to generate the estimated coefficient vectors, ${\hat{\beta }}_{j},\;j=1,\;2,\;\ldots ,\;500$ (see SVRS algorithm steps above).

**Table 2:** Sample size (*n*) of the full dataset generated under each class-imbalance ratio (*I*_*R*_) to achieve a target balanced sample size (*n*_*b*_).

*n*_*b*_	*I*_*R*_		
	1:50	1:100	1:1000
1000	25,500	50,500	500,500
2000	51,000	101,000	1,001,000
3000	76,500	151,500	1,501,500
4000	102,000	202,000	2,002,000
5000	127,500	252,500	2,502,500

**Fig 1:**
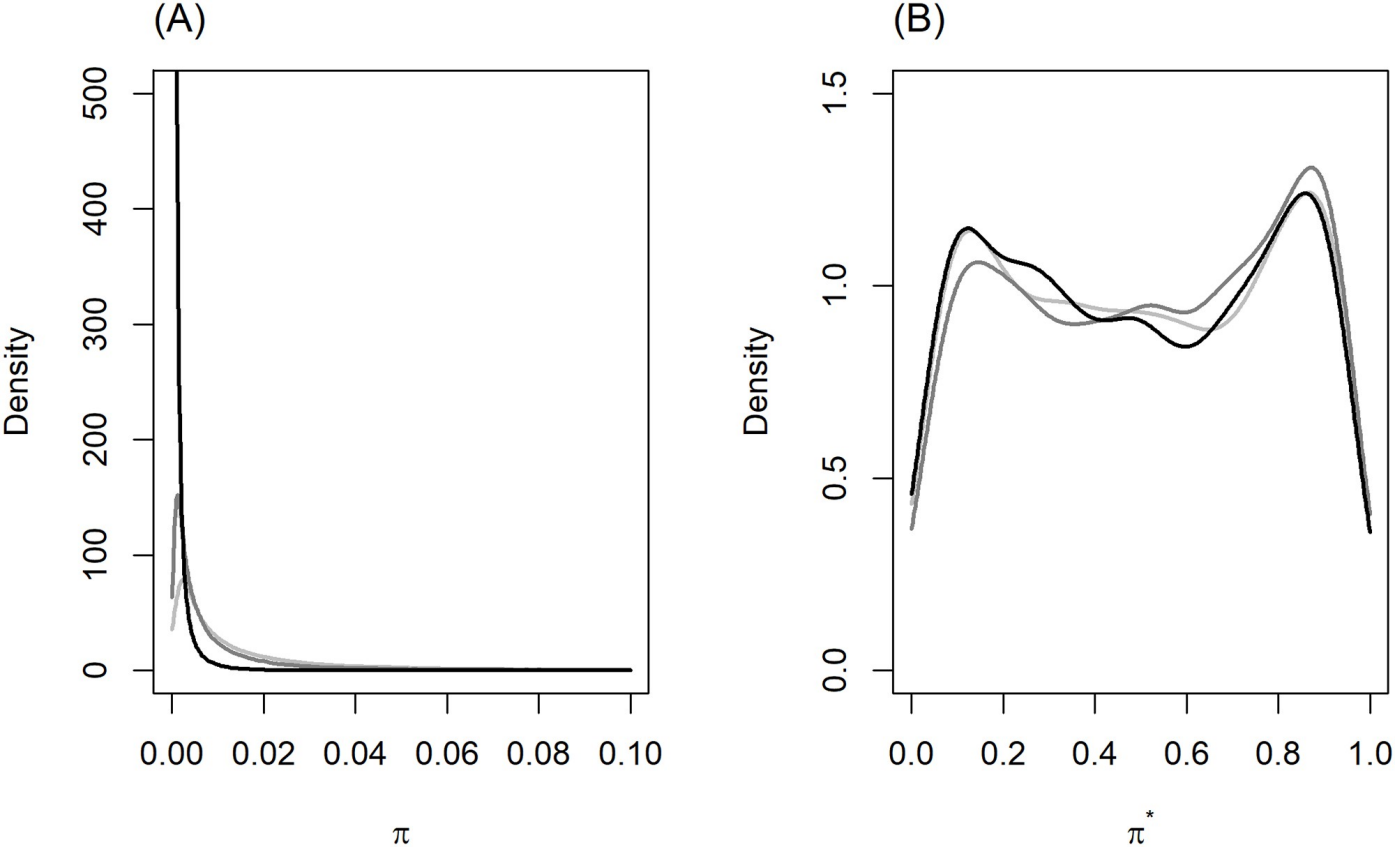
Distribution of simulated probabilities with imbalance ratios 1:50 (black), 1:100 (dark grey), and 1:1000 (light grey), under COR-24. (A) Original imbalanced datasets (B) Balanced datasets based on response-based downsampling.

Fig 1 provides an example of the distribution of simulated probabilities of the positive class in the original data sample (of size *n*) and for balanced datasets under COR-24 simulation model with varying imbalance ratios (*I*_*R*_). We notice that distribution of simulated probability for the original imbalanced data is highly skewed towards zero (i.e. cases are rare), where degree of skewness exacerbates as *I*_*R*_ becomes severe (Fig 1A). However, balancing the sample removes the skewness and renders a distribution that is invariant across imbalance ratios (Fig 1B). We observed similar characteristics in other simulation models as well.

## Results

**Fig 2:**
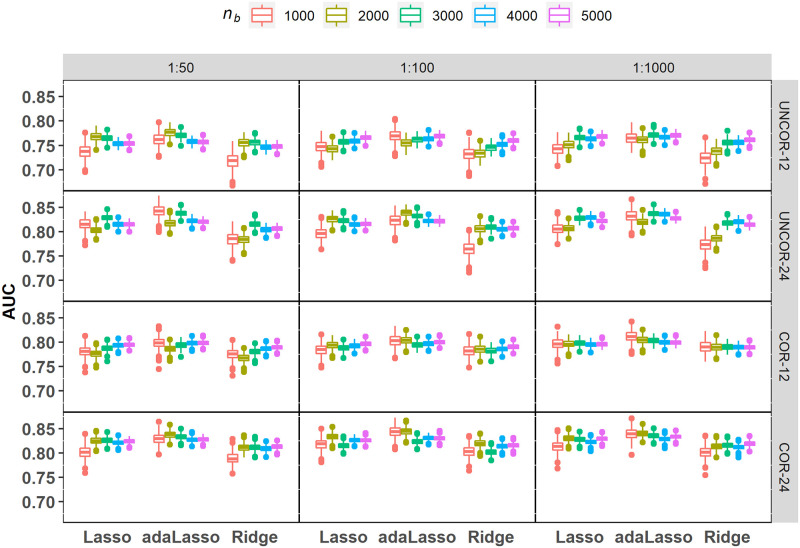
Predictive performance for various regularized logistic regression models fitted to simulated balanced datasets with imbalance ratios 1:50, 1:100 and 1:1000. AUC values reported here correspond to *λ*_*min*_.

The simulation results in this section focus on variable selection performance of the SVRS algorithm based on performance metrics such as true positive rate (TPR), false positive rate (FPR), false discovery rate (FDR) and area under the ROC curve (AUC). The rate metrics are defined as follows: *TPR* = *TP*/*s*; *FPR* = *FP*/*m* and *FDR* = *FP*/(*TP* + *FP*), where *s*, *m*, *FP* and *TP* denote number of signal covariates, noise covariates, false positives, and true positives, respectively. We also compare selection performance with the distribution of these metrics computed from variable selection performed on the 500 individual fits of Lasso and adaLasso where variables with non-zero regression coefficients are retained in the selected set. Notice that ridge regression does not perform automatic variable selection and therefore selection performance based on individual fits is presented for Lasso and adaLasso methods only. Fig 2 reports a summary of the predictive performance of the fitted regularized logistic regression models across all simulation scenarios. We find that the individual model fits have reasonable skill in predicting the binary response variable based on the mean cross-validated AUC scores corresponding to the optimal value of the tuning parameter, denoted here as *λ*_*min*_. Notice that all results presented in this section are based on *λ*_*min*_.

### Selection performance with un-correlated data

#### UNCOR-12

**Fig 3:**
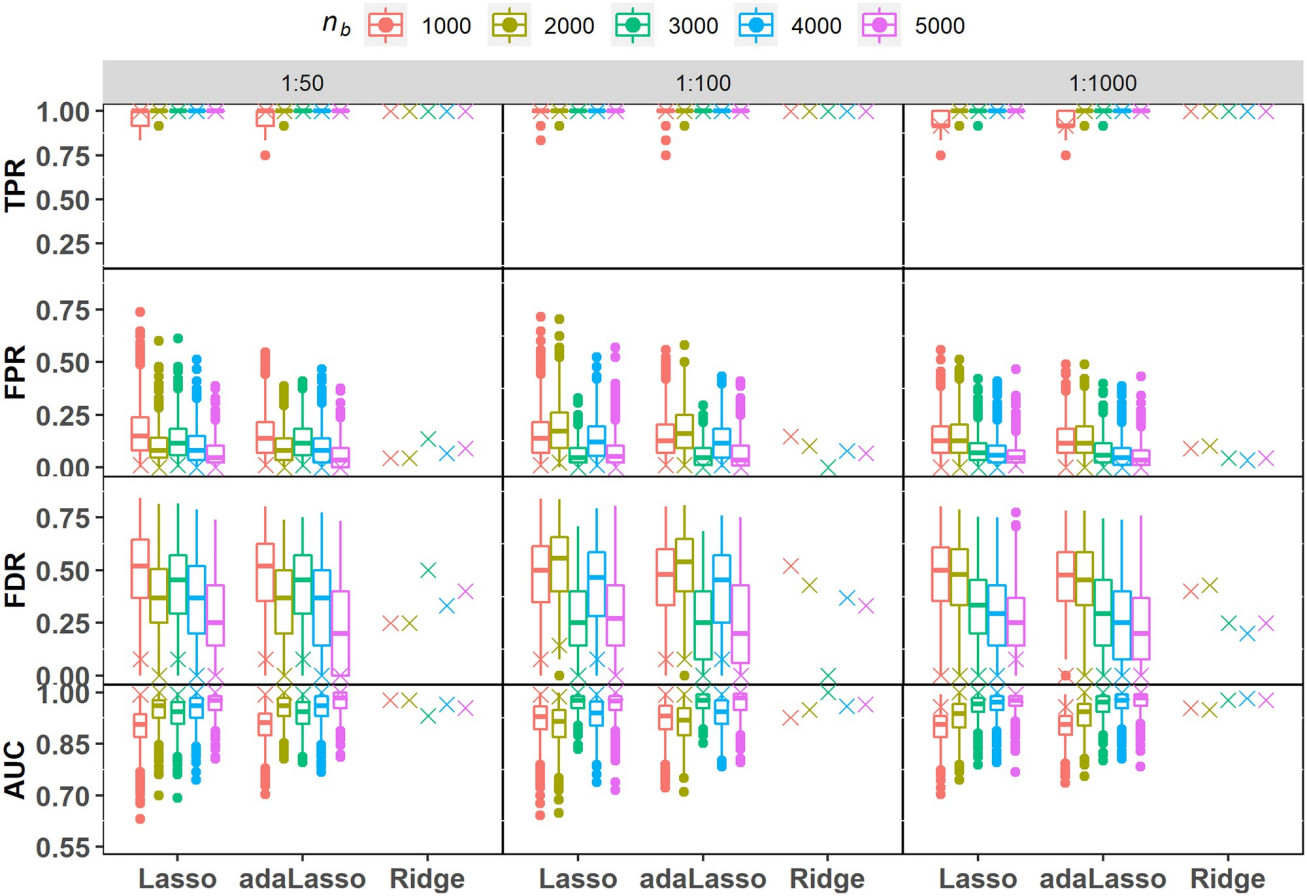
Simulation under UNCOR-12. Performance metric scores for variable selection based on the SVRS algorithm (symbol x) and corresponding distributions across 500 individual fits of each regularization model with imbalance ratios 1:50, 1:100 and 1:1000. Performance scores for individual ridge regression fits are not applicable as it does not perform automatic variable selection.

Fig 3 depicts TPR, FPR, FDR and AUC values obtained from the SVRS algorithm and corresponding distribution of scores across the individual fits for the various regularization methods, imbalance ratios (*I*_*R*_) and balanced sample sizes, *n*_*b*_. Apart from the considerable variation in individual TPR scores for Lasso at the smallest sample size (*n*_*b*_ = 1000), both Lasso and adaLasso scores are stable and close to 1 irrespective of the sample size and *I*_*R*_. It is also evident that SVRS TPR scores are near perfect for the three regularization methods in all scenarios. The individual FPR and FDR scores for Lasso and ada-Lasso are however much more variable with median values dropping as a function of *n*_*b*_. The highest mean FPR score for the Lasso and adaLasso was 0.191 and 0.176 respectively, which is approximately 17 and 15 noise covariates selected on average (Table 3). Similarly, the maximum FDR values ranged from 0.261 to 0.521, and 0.218 to 0.504 for Lasso and adaLasso respectively, showing that a substantially large proportion of selected covariates from an individual fit can be false positives. The SVRS FPR and FDR scores across the two Lasso methods on the other hand range from 0 to 0.023, and 0 to 0.143 respectively, across all scenarios (Table 3). In comparison, ridge regression registers higher SVRS FPR and FDR values ranging in 0 to 0.136 and 0 to 0.520 respectively.

**Table 3:** Simulation under UNCOR-12. Performance metric scores for variable selection based on the SVRS algorithm and average scores (reported in parenthesis) across 500 individual fits of each regularization model with imbalance ratios 1:50, 1:100 and 1:1000. Performance scores for individual ridge regression fits are not applicable as it does not perform automatic variable selection.

Performance Metric	*n*_*b*_	Lasso			adaLasso			Ridge		
		1:50	1:100	1:1000	1:50	1:100	1:1000	1:50	1:100	1:1000
TPR	1000	1.000	1.000	0.917	1.000	1.000	0.917	1.000	1.000	1.000
		(*0*.*965*)	(*0*.*991*)	(*0*.*938*)	(*0*.*963*)	(*0*.*990*)	(*0*.*936*)			
	2000	1.000	1.000	1.000	1.000	1.000	1.000	1.000	1.000	1.000
		(*1*.*000*)	(*0*.*998*)	(*1*.*000*)	(*1*.*000*)	(*0*.*997*)	(*1*.*000*)			
	3000	1.000	1.000	1.000	1.000	1.000	1.000	1.000	1.000	1.000
		(*1*.*000*)	(*1*.*000*)	(*1*.*000*)	(*1*.*000*)	(*1*.*000*)	(*1*.*000*)			
	4000	1.000	1.000	1.000	1.000	1.000	1.000	1.000	1.000	1.000
		(*1*.*000*)	(*1*.*000*)	(*1*.*000*)	(*1*.*000*)	(*1*.*000*)	(*1*.*000*)			
	5000	1.000	1.000	1.000	1.000	1.000	1.000	1.000	1.000	1.000
		(*1*.*000*)	(*1*.*000*)	(*1*.*000*)	(*1*.*000*)	(*1*.*000*)	(*1*.*000*)			
FPR	1000	0.011	0.011	0.000	0.011	0.011	0.000	0.045	0.148	0.091
		(*0*.*172*)	(*0*.*160*)	(*0*.*143*)	(*0*.*158*)	(*0*.*148*)	(*0*.*134*)			
	2000	0.000	0.023	0.000	0.000	0.011	0.000	0.045	0.102	0.102
		(*0*.*102*)	(*0*.*191*)	(*0*.*145*)	(*0*.*093*)	(*0*.*176*)	(*0*.*135*)			
	3000	0.011	0.000	0.000	0.011	0.000	0.000	0.136	0.000	0.045
		(*0*.*136*)	(*0*.*067*)	(*0*.*086*)	(*0*.*124*)	(*0*.*058*)	(*0*.*077*)			
	4000	0.000	0.011	0.000	0.000	0.011	0.000	0.068	0.080	0.034
		(*0*.*104*)	(*0*.*137*)	(*0*.*074*)	(*0*.*095*)	(*0*.*124*)	(*0*.*066*)			
	5000	0.000	0.000	0.011	0.000	0.000	0.000	0.091	0.068	0.045
		(*0*.*066*)	(*0*.*076*)	(*0*.*061*)	(*0*.*058*)	(*0*.*066*)	(*0*.*051*)			
FDR	1000	0.077	0.077	0.000	0.077	0.077	0.000	0.250	0.520	0.400
		(*0*.*498*)	(*0*.*475*)	(*0*.*473*)	(*0*.*482*)	(*0*.*460*)	(*0*.*460*)			
	2000	0.000	0.143	0.000	0.000	0.077	0.000	0.250	0.429	0.429
		(*0*.*372*)	(*0*.*521*)	(*0*.*460*)	(*0*.*349*)	(*0*.*504*)	(*0*.*443*)			
	3000	0.077	0.000	0.000	0.077	0.000	0.000	0.500	0.000	0.250
		(*0*.*440*)	(*0*.*284*)	(*0*.*331*)	(*0*.*419*)	(*0*.*252*)	(*0*.*301*)			
	4000	0.000	0.077	0.000	0.000	0.077	0.000	0.333	0.368	0.200
		(*0*.*368*)	(*0*.*430*)	(*0*.*290*)	(*0*.*339*)	(*0*.*404*)	(*0*.*257*)			
	5000	0.000	0.000	0.077	0.000	0.000	0.000	0.400	0.333	0.250
		(*0*.*266*)	(*0*.*288*)	(*0*.*262*)	(*0*.*232*)	(*0*.*252*)	(*0*.*218*)			
AUC	1000	0.994	0.994	0.958	0.994	0.994	0.958	0.977	0.926	0.955
		(*0*.*896*)	(*0*.*916*)	(*0*.*897*)	(*0*.*902*)	(*0*.*921*)	(*0*.*901*)			
	2000	1.000	0.989	1.000	1.000	0.994	1.000	0.977	0.949	0.949
		(*0*.*949*)	(*0*.*903*)	(*0*.*927*)	(*0*.*953*)	(*0*.*911*)	(*0*.*932*)			
	3000	0.994	1.000	1.000	0.994	1.000	1.000	0.932	1.000	0.977
		(*0*.*932*)	(*0*.*967*)	(*0*.*957*)	(*0*.*938*)	(*0*.*971*)	(*0*.*961*)			
	4000	1.000	0.994	1.000	1.000	0.994	1.000	0.966	0.960	0.983
		(*0*.*948*)	(*0*.*932*)	(*0*.*963*)	(*0*.*953*)	(*0*.*938*)	(*0*.*967*)			
	5000	1.000	1.000	0.994	1.000	1.000	1.000	0.955	0.966	0.977
		(*0*.*967*)	(*0*.*962*)	(*0*.*969*)	(*0*.*971*)	(*0*.*967*)	(*0*.*974*)			

The suboptimal performance of the individual across Lasso and adaLasso fits is also clear from viewing the large variability in AUC scores (Fig 3), with poor scores observed in all scenarios. SVRS algorithm improves the selection performance by a substantial margin with AUC scores ranging in 0.958 to 1 for the two Lasso methods. SVRS based AUC scores for Ridge regression are comparatively lower due mostly to worse performance in terms of FPR and FDR.

#### UNCOR-24

**Fig 4:**
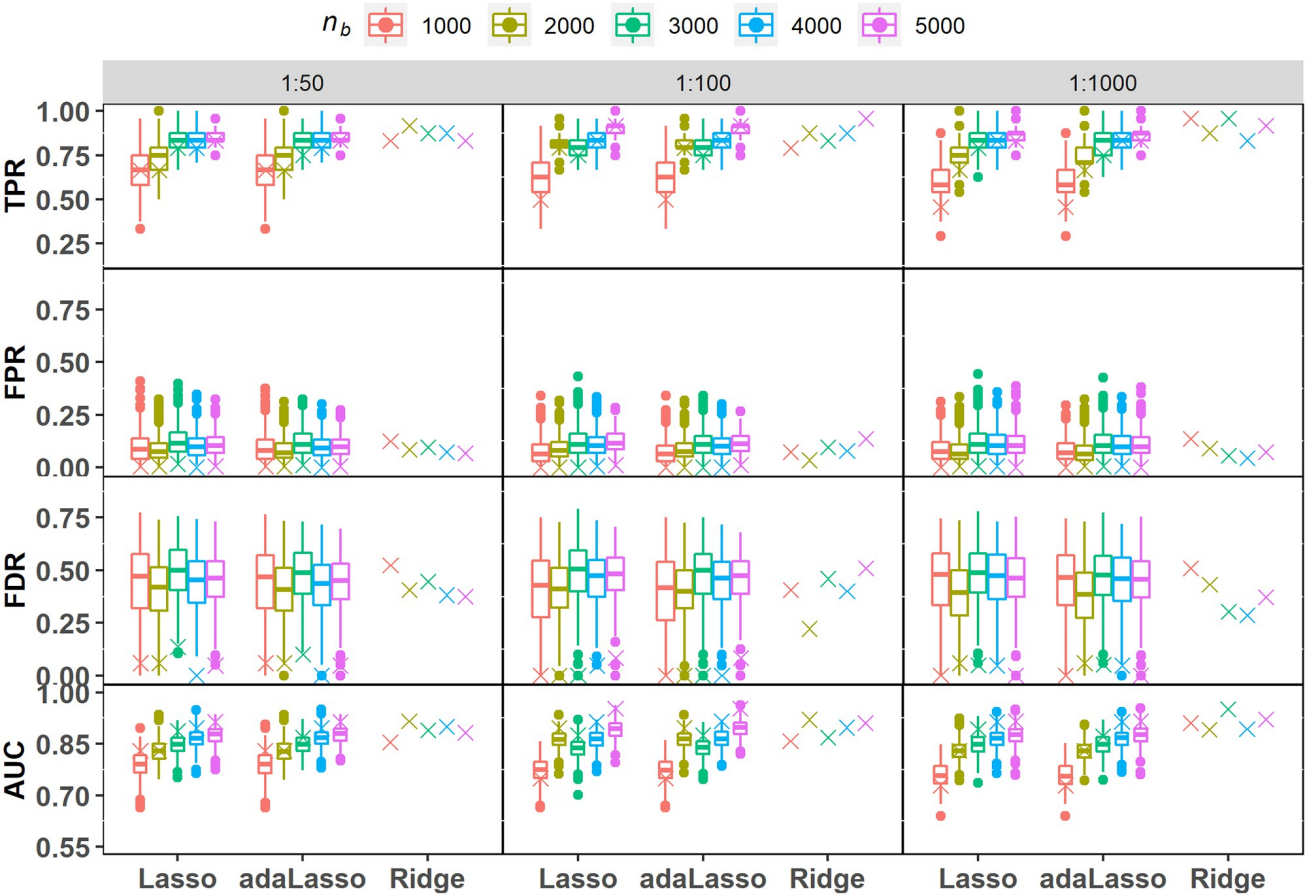
Simulation under UNCOR-24. Performance metric scores for variable selection based on the SVRS algorithm (symbol x) and corresponding distributions across 500 individual fits of each regularization model with imbalance ratios 1:50, 1:100 and 1:1000. Performance scores for individual ridge regression fits are not applicable as it does not perform automatic variable selection.

Distributions of TPR values for the two Lasso methods reveal that individual fits tend to recover a much lower proportion of signal covariates under UNCOR-24 when compared to UNCOR-12; however, mean TPR increases with sample size (Fig 4; Table 4). This is explained in part from noticing that UNCOR-24 has a substantial proportion of covariates with relatively small effect sizes (|*β*_*i*_| < 0.35; Table 1) that are hard to detect at a lower sample size. The SVRS TPR scores on the other hand are generally above the 25^th^ percentile of the individual scores, especially for higher sample sizes. Ridge regression shows further improvement in terms of SVRS TPR scores. Behavior of FPR and FDR scores based on individual fits and SVRS algorithm is similar to that of under UNCOR-12 with one exception: scores do not improve much with the increasing sampling size. It is evident from Fig 4 that SVRS AUC scores are generally higher than the median score from the individual fits, which shows that SVRS is superior at achieving stable ranking and selection as compared to individual fits.

**Table 4:** Simulation under UNCOR-24. Performance metric scores for variable selection based on the SVRS algorithm and average scores (reported in parenthesis) across 500 individual fits of each regularization model with imbalance ratios 1:50, 1:100 and 1:1000. Performance scores for individual ridge regression fits are not applicable as it does not perform automatic variable selection.

Performance Metric	*n*_*b*_	Lasso			adaLasso			Ridge		
		1:50	1:100	1:1000	1:50	1:100	1:1000	1:50	1:100	1:1000
TPR	1000	0.667	0.500	0.458	0.667	0.500	0.458	0.833	0.792	0.958
		(*0*.*674*)	(*0*.*626*)	(*0*.*604*)	(*0*.*671*)	(*0*.*622*)	(*0*.*600*)			
	2000	0.667	0.792	0.667	0.667	0.792	0.667	0.917	0.875	0.875
		(*0*.*740*)	(*0*.*813*)	(*0*.*737*)	(*0*.*737*)	(*0*.*811*)	(*0*.*733*)			
	3000	0.792	0.750	0.792	0.750	0.750	0.750	0.875	0.833	0.958
		(*0*.*819*)	(*0*.*789*)	(*0*.*816*)	(*0*.*815*)	(*0*.*787*)	(*0*.*811*)			
	4000	0.792	0.833	0.833	0.792	0.833	0.833	0.875	0.875	0.833
		(*0*.*834*)	(*0*.*834*)	(*0*.*841*)	(*0*.*832*)	(*0*.*832*)	(*0*.*838*)			
	5000	0.833	0.917	0.833	0.833	0.917	0.833	0.833	0.958	0.917
		(*0*.*856*)	(*0*.*904*)	(*0*.*863*)	(*0*.*854*)	(*0*.*903*)	(*0*.*860*)			
FPR	1000	0.006	0.000	0.000	0.006	0.000	0.000	0.125	0.074	0.136
		(*0*.*172*)	(*0*.*160*)	(*0*.*143*)	(*0*.*092*)	(*0*.*075*)	(*0*.*082*)			
	2000	0.006	0.000	0.006	0.006	0.000	0.006	0.085	0.034	0.091
		(*0*.*102*)	(*0*.*191*)	(*0*.*145*)	(*0*.*081*)	(*0*.*086*)	(*0*.*075*)			
	3000	0.017	0.000	0.006	0.011	0.000	0.006	0.097	0.097	0.057
		(*0*.*136*)	(*0*.*067*)	(*0*.*086*)	(*0*.*118*)	(*0*.*113*)	(*0*.*114*)			
	4000	0.000	0.006	0.006	0.000	0.000	0.006	0.074	0.080	0.045
		(*0*.*104*)	(*0*.*137*)	(*0*.*074*)	(*0*.*097*)	(*0*.*104*)	(*0*.*108*)			
	5000	0.006	0.011	0.000	0.006	0.011	0.000	0.068	0.136	0.074
		(*0*.*066*)	(*0*.*076*)	(*0*.*061*)	(*0*.*103*)	(*0*.*114*)	(*0*.*108*)			
FDR	1000	0.059	0.000	0.000	0.059	0.000	0.000	0.524	0.406	0.511
		(*0*.*439*)	(*0*.*405*)	(*0*.*447*)	(*0*.*432*)	(*0*.*399*)	(*0*.*440*)			
	2000	0.059	0.000	0.059	0.059	0.000	0.059	0.405	0.222	0.432
		(*0*.*410*)	(*0*.*413*)	(*0*.*388*)	(*0*.*403*)	(*0*.*402*)	(*0*.*380*)			
	3000	0.136	0.000	0.050	0.100	0.000	0.053	0.447	0.459	0.303
		(*0*.*493*)	(*0*.*485*)	(*0*.*479*)	(*0*.*482*)	(*0*.*474*)	(*0*.*468*)			
	4000	0.000	0.048	0.048	0.000	0.000	0.048	0.382	0.400	0.286
		(*0*.*441*)	(*0*.*458*)	(*0*.*460*)	(*0*.*428*)	(*0*.*446*)	(*0*.*447*)			
	5000	0.048	0.083	0.000	0.048	0.083	0.000	0.375	0.511	0.371
		(*0*.*453*)	(*0*.*472*)	(*0*.*459*)	(*0*.*440*)	(*0*.*457*)	(*0*.*447*)			
AUC	1000	0.830	0.750	0.729	0.830	0.750	0.729	0.854	0.859	0.911
		(*0*.*789*)	(*0*.*774*)	(*0*.*759*)	(*0*.*789*)	(*0*.*773*)	(*0*.*759*)			
	2000	0.830	0.896	0.830	0.830	0.896	0.830	0.916	0.920	0.892
		(*0*.*828*)	(*0*.*862*)	(*0*.*830*)	(*0*.*828*)	(*0*.*863*)	(*0*.*829*)			
	3000	0.887	0.875	0.893	0.869	0.875	0.872	0.889	0.868	0.951
		(*0*.*848*)	(*0*.*835*)	(*0*.*848*)	(*0*.*849*)	(*0*.*837*)	(*0*.*848*)			
	4000	0.896	0.914	0.914	0.896	0.917	0.914	0.901	0.898	0.894
		(*0*.*865*)	(*0*.*862*)	(*0*.*863*)	(*0*.*867*)	(*0*.*864*)	(*0*.*865*)			
	5000	0.914	0.953	0.917	0.914	0.953	0.917	0.883	0.911	0.921
		(*0*.*873*)	(*0*.*892*)	(*0*.*874*)	(*0*.*876*)	(*0*.*895*)	(*0*.*876*)			

### Selection performance with correlated data

#### COR-12

**Fig 5:**
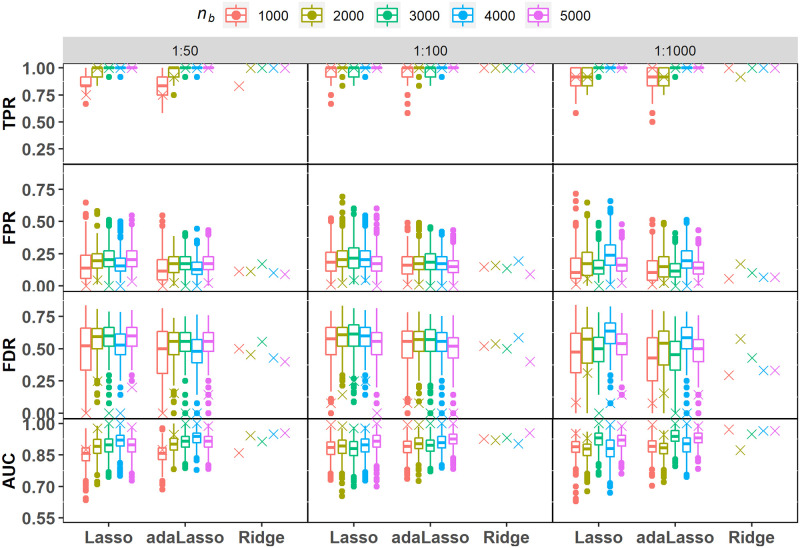
Simulation under COR-12. Performance metric scores for variable selection based on the SVRS algorithm (symbol x) and corresponding distributions across 500 individual fits of each regularization model with imbalance ratios 1:50, 1:100 and 1:1000. Performance scores for individual ridge regression fits are not applicable as it does not perform automatic variable selection.

Mean TPR scores under COR-12 in individual fits of the various regularization methods are lower as compared to those of attained under UNCOR-12, falling in the range of 0.847 to 1 (Table 5). Likewise, SVRS tends to recover most or all signal covariates despite the severe class-imbalance and high correlation between the signal and noise covariates (TPR > 0.83 for *n*_*b*_ > 1000; Table 5). Even at *n*_*b*_ = 1000, SVRS TPR values are higher than the 25^th^ percentile of the individual score distributions in most cases (Fig 5). Performance in terms of FPR and FDR is again similar to the pattern seen under the un-correlated simulation models as individual Lasso and adaLasso scores are much worse with high variability as compared to SVRS scores. Ridge regression has substantially higher SVRS FPR and FDR scores. We see that the two Lasso methods have similar performance in terms of AUC scores, which is far superior as compared to the individual fits, with values range from 0.875 to 1 across the two methods. On the other hand, SVRS AUC scores for ridge regression are relatively smaller owing to large number of higher false positive discoveries.

**Table 5:** Simulation under COR-12. Performance metric scores for variable selection based on the SVRS algorithm and average scores (reported in parenthesis) across 500 individual fits of each regularization model with imbalance ratios 1:50, 1:100 and 1:1000. Performance scores for individual ridge regression fits are not applicable as it does not perform automatic variable selection.

Performance Metric	*n*_*b*_	Lasso			adaLasso			Ridge		
		1:50	1:100	1:1000	1:50	1:100	1:1000	1:50	1:100	1:1000
TPR	1000	0.750	1.000	0.917	0.750	1.000	1.000	0.833	1.000	1.000
		(*0*.*862*)	(*0*.*949*)	(*0*.*911*)	(*0*.*847*)	(*0*.*939*)	(*0*.*896*)			
	2000	1.000	1.000	0.917	0.917	1.000	0.917	1.000	1.000	0.917
		(*0*.*974*)	(*0*.*989*)	(*0*.*921*)	(*0*.*965*)	(*0*.*985*)	(*0*.*916*)			
	3000	1.000	1.000	1.000	1.000	1.000	1.000	1.000	1.000	1.000
		(*1*.*000*)	(*0*.*974*)	(*0*.*999*)	(*0*.*999*)	(*0*.*970*)	(*0*.*998*)			
	4000	1.000	1.000	1.000	1.000	1.000	1.000	1.000	1.000	1.000
		(*1*.*000*)	(*0*.*999*)	(*1*.*000*)	(*1*.*000*)	(*0*.*997*)	(*1*.*000*)			
	5000	1.000	1.000	1.000	1.000	1.000	1.000	1.000	1.000	1.000
		(*1*.*000*)	(*1*.*000*)	(*1*.*000*)	(*1*.*000*)	(*1*.*000*)	(*1*.*000*)			
FPR	1000	0.000	0.011	0.011	0.000	0.011	0.011	0.114	0.148	0.057
		(*0*.*160*)	(*0*.*192*)	(*0*.*146*)	(*0*.*138*)	(*0*.*166*)	(*0*.*122*)			
	2000	0.045	0.023	0.057	0.023	0.011	0.023	0.114	0.159	0.170
		(*0*.*199*)	(*0*.*218*)	(*0*.*181*)	(*0*.*165*)	(*0*.*182*)	(*0*.*160*)			
	3000	0.000	0.045	0.000	0.000	0.000	0.000	0.170	0.136	0.102
		(*0*.*215*)	(*0*.*220*)	(*0*.*151*)	(*0*.*178*)	(*0*.*184*)	(*0*.*126*)			
	4000	0.000	0.045	0.011	0.000	0.000	0.000	0.102	0.193	0.068
		(*0*.*170*)	(*0*.*215*)	(*0*.*251*)	(*0*.*136*)	(*0*.*178*)	(*0*.*210*)			
	5000	0.034	0.000	0.023	0.023	0.000	0.023	0.091	0.091	0.068
		(*0*.*215*)	(*0*.*185*)	(*0*.*169*)	(*0*.*174*)	(*0*.*153*)	(*0*.*140*)			
FDR	1000	0.000	0.077	0.083	0.000	0.077	0.077	0.500	0.520	0.294
		(*0*.*486*)	(*0*.*540*)	(*0*.*449*)	(*0*.*461*)	(*0*.*510*)	(*0*.*414*)			
	2000	0.250	0.143	0.313	0.154	0.077	0.154	0.455	0.538	0.577
		(*0*.*566*)	(*0*.*585*)	(*0*.*517*)	(*0*.*524*)	(*0*.*543*)	(*0*.*489*)			
	3000	0.000	0.250	0.000	0.000	0.000	0.000	0.556	0.500	0.429
		(*0*.*582*)	(*0*.*581*)	(*0*.*489*)	(*0*.*538*)	(*0*.*543*)	(*0*.*440*)			
	4000	0.000	0.250	0.077	0.000	0.000	0.000	0.429	0.586	0.333
		(*0*.*523*)	(*0*.*582*)	(*0*.*614*)	(*0*.*465*)	(*0*.*535*)	(*0*.*574*)			
	5000	0.200	0.000	0.143	0.143	0.000	0.143	0.400	0.400	0.333
		(*0*.*586*)	(*0*.*541*)	(*0*.*523*)	(*0*.*534*)	(*0*.*493*)	(*0*.*468*)			
AUC	1000	0.875	0.994	0.953	0.875	0.994	0.994	0.860	0.926	0.972
		(*0*.*851*)	(*0*.*879*)	(*0*.*883*)	(*0*.*855*)	(*0*.*886*)	(*0*.*887*)			
	2000	0.977	0.989	0.930	0.947	0.994	0.947	0.943	0.920	0.873
		(*0*.*888*)	(*0*.*885*)	(*0*.*870*)	(*0*.*900*)	(*0*.*902*)	(*0*.*878*)			
	3000	1.000	0.977	1.000	1.000	1.000	1.000	0.915	0.932	0.949
		(*0*.*893*)	(*0*.*877*)	(*0*.*924*)	(*0*.*911*)	(*0*.*893*)	(*0*.*936*)			
	4000	1.000	0.977	0.994	1.000	1.000	1.000	0.949	0.903	0.966
		(*0*.*915*)	(*0*.*892*)	(*0*.*874*)	(*0*.*932*)	(*0*.*910*)	(*0*.*895*)			
	5000	0.983	1.000	0.989	0.989	1.000	0.989	0.955	0.955	0.966
		(*0*.*892*)	(*0*.*907*)	(*0*.*915*)	(*0*.*913*)	(*0*.*923*)	(*0*.*930*)			

#### COR-24

**Fig 6:**
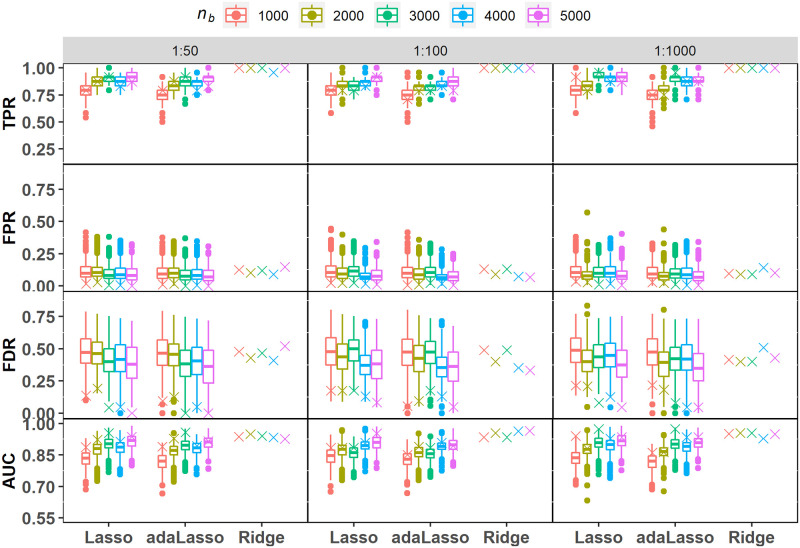
Simulation under COR-24. Performance metric scores for variable selection based on the SVRS algorithm (symbol x) and corresponding distributions across 500 individual fits of each regularization model with imbalance ratios 1:50, 1:100 and 1:1000. Performance scores for individual ridge regression fits are not applicable as it does not perform automatic variable selection.

COR-24 is the most involved simulation model including several highly correlated candidate covariates (Table 1) where the correlation structure spans across signal and noise variables. Here we see that the mean TPR scores for Lasso/adaLasso individual fits range from 0.740 to 0.921 with high variability in the score distribution (Fig 6). On the other hand, SVRS based TPR values exceed the 25^th^ percentile of the individual score distribution in most cases. It is evident that SVRS algorithm with Lasso/adaLasso excels at filtering out the noise covariates with very low FPR and FDR scores ranging from 0 to 0.034 and 0 to 0.217, respectively. Individual Lasso/adaLasso fits in comparison do not provide a reliable selection tool as they tend to include a very large proportion of noise covariates in the selection set (Fig 6; Table 6; FDR range: 0.349 to 0.485). Ridge regression has near perfect SVRS TPR values in all cases, but with much higher FPR and FDR scores as compared to Lasso and adaLasso. However, SVRS based selection performance of the three regularization methods is quite similar in terms of AUC scores with Ridge regression registering slightly higher scores (Fig 6; Table 6). We emphasize that a majority of SVRS AUC scores for Lasso and adaLasso lie beyond the 75^th^ percentile of the individual score distributions across all scenarios. Overall, our results clearly demonstrate that SVRS algorithm renders a marked improvement over individual fits by stabilising the selection variability inherent in those fits.

**Table 6:** Simulation under COR-24. Performance metric scores for variable selection based on the SVRS algorithm and average scores (reported in parenthesis) across 500 individual fits of each regularization model with imbalance ratios 1:50, 1:100 and 1:1000. Performance scores for individual ridge regression fits are not applicable as it does not perform automatic variable selection.

Performance Metric	*n*_*b*_	Lasso			adaLasso			Ridge		
		1:50	1:100	1:1000	1:50	1:100	1:1000	1:50	1:100	1:1000
TPR	1000	0.792	0.792	0.917	0.792	0.708	0.750	1.000	1.000	1.000
		(*0*.*778*)	(*0*.*802*)	(*0*.*787*)	(*0*.*743*)	(*0*.*763*)	(*0*.*740*)			
	2000	0.875	0.792	0.792	0.875	0.792	0.750	1.000	1.000	1.000
		(*0*.*867*)	(*0*.*848*)	(*0*.*840*)	(*0*.*842*)	(*0*.*817*)	(*0*.*805*)			
	3000	0.917	0.792	0.958	0.917	0.792	0.958	1.000	1.000	1.000
		(*0*.*897*)	(*0*.*839*)	(*0*.*921*)	(*0*.*872*)	(*0*.*817*)	(*0*.*898*)			
	4000	0.833	0.833	0.875	0.792	0.833	0.875	0.958	1.000	1.000
		(*0*.*868*)	(*0*.*867*)	(*0*.*899*)	(*0*.*854*)	(*0*.*850*)	(*0*.*873*)			
	5000	0.875	0.917	0.875	0.833	0.792	0.875	1.000	1.000	1.000
		(*0*.*919*)	(*0*.*902*)	(*0*.*918*)	(*0*.*897*)	(*0*.*872*)	(*0*.*889*)			
FPR	1000	0.017	0.023	0.034	0.011	0.006	0.028	0.125	0.131	0.097
		(*0*.*113*)	(*0*.*116*)	(*0*.*117*)	(*0*.*104*)	(*0*.*107*)	(*0*.*105*)			
	2000	0.028	0.023	0.028	0.017	0.011	0.023	0.102	0.091	0.091
		(*0*.*117*)	(*0*.*105*)	(*0*.*087*)	(*0*.*107*)	(*0*.*096*)	(*0*.*077*)			
	3000	0.006	0.023	0.011	0.000	0.023	0.011	0.119	0.131	0.091
		(*0*.*095*)	(*0*.*120*)	(*0*.*110*)	(*0*.*085*)	(*0*.*109*)	(*0*.*100*)			
	4000	0.006	0.017	0.017	0.006	0.017	0.006	0.091	0.074	0.142
		(*0*.*101*)	(*0*.*079*)	(*0*.*111*)	(*0*.*093*)	(*0*.*071*)	(*0*.*099*)			
	5000	0.000	0.011	0.006	0.000	0.006	0.006	0.148	0.068	0.102
		(*0*.*092*)	(*0*.*087*)	(*0*.*088*)	(*0*.*082*)	(*0*.*078*)	(*0*.*077*)			
FDR	1000	0.136	0.174	0.214	0.095	0.056	0.217	0.478	0.489	0.415
		(*0*.*478*)	(*0*.*472*)	(*0*.*485*)	(*0*.*465*)	(*0*.*463*)	(*0*.*470*)			
	2000	0.192	0.174	0.208	0.125	0.095	0.182	0.429	0.400	0.400
		(*0*.*467*)	(*0*.*440*)	(*0*.*404*)	(*0*.*447*)	(*0*.*422*)	(*0*.*379*)			
	3000	0.043	0.174	0.080	0.000	0.174	0.080	0.467	0.489	0.400
		(*0*.*407*)	(*0*.*485*)	(*0*.*439*)	(*0*.*380*)	(*0*.*463*)	(*0*.*418*)			
	4000	0.048	0.130	0.125	0.050	0.130	0.045	0.410	0.351	0.510
		(*0*.*422*)	(*0*.*378*)	(*0*.*442*)	(*0*.*406*)	(*0*.*353*)	(*0*.*415*)			
	5000	0.000	0.083	0.045	0.000	0.050	0.045	0.520	0.333	0.429
		(*0*.*384*)	(*0*.*380*)	(*0*.*380*)	(*0*.*357*)	(*0*.*356*)	(*0*.*349*)			
AUC	1000	0.887	0.884	0.941	0.890	0.851	0.861	0.938	0.935	0.952
		(*0*.*832*)	(*0*.*843*)	(*0*.*835*)	(*0*.*820*)	(*0*.*828*)	(*0*.*817*)			
	2000	0.923	0.884	0.882	0.929	0.890	0.864	0.949	0.955	0.955
		(*0*.*875*)	(*0*.*871*)	(*0*.*877*)	(*0*.*868*)	(*0*.*861*)	(*0*.*864*)			
	3000	0.955	0.884	0.973	0.958	0.884	0.973	0.940	0.935	0.955
		(*0*.*901*)	(*0*.*859*)	(*0*.*905*)	(*0*.*893*)	(*0*.*854*)	(*0*.*899*)			
	4000	0.914	0.908	0.929	0.893	0.908	0.935	0.934	0.963	0.929
		(*0*.*884*)	(*0*.*894*)	(*0*.*894*)	(*0*.*880*)	(*0*.*890*)	(*0*.*887*)			
	5000	0.938	0.953	0.935	0.917	0.893	0.935	0.926	0.966	0.949
		(*0*.*914*)	(*0*.*908*)	(*0*.*915*)	(*0*.*908*)	(*0*.*897*)	(*0*.*906*)			

### Case study: Analysis of wildland fire occurrence data

In this section, we consider a large wildland fire dataset compiled by Nadeem et al. [29] comprising fire occurrence records by ignition source (human- and lightning-caused) and a suite of explanatory variables on a fine spatial grid in British Columbia, Canada, during 1981–2014. Wildland fires account for an average of 2.5 million hectares of burnt area across Canada annually, costing the government of Canada up to $1.5 billion per year in fire suppression and management [43]. The province of British Columbia is a major contributor to daily fire load in Canada, as 70% of its land area contains coniferous forest or grasslands that are potentially flammable [29]. Anthropogenic climate change is attributed to further exacerbate the risk of extreme fire-weather conditions in recent years [44]. For instance, a record 1.2 million ha of wildland burned during the historically extreme 2017 wildfire season alone [45]. It is therefore crucial to develop robust statistical models that enable identification of key underlying drivers of fire occurrence process and predicting daily ignitions across the province for fire management preparedness and detection planning.

Response variable in Nadeem et al.’s [29] dataset is a Bernoulli random variable *Y* (indicator variable for at least one ignition) observed on a space-time voxel that represents a 24-h time period for a 20×20-km cell in the National Forest Inventory (NFI) grid [46]. There are approximately 13 million 1-day space–time voxels spanning 2541 grid cells and 34 fire seasons (1981–2014) where human- and lightning-caused fire occurrences were observed in only 0.23% and 0.18% of the voxels respectively, which highlights the severity of class-imbalance in the observed distribution of *Y*. A large number of candidate covariates were compiled including geographic, vegetation, ecumene, surface fire weather, atmospheric stability and other derived variables (see Table 1 in Nadeem et al. [29]). We refer the reader to Nadeem et al. [29] for more details about the study area and methods used in data collection and compilation.

Nadeem et al. [29] employed the lasso-logistic modelling framework to develop the following three fire occurrence models: i) a human-caused fire (HCF) model; ii) a lightning-caused fire (OLCF) model which included *observed* cloud-to-ground lightning strikes as a candidate covariate, and iii) a lightning-caused fire (PLCF) model which did not include the lightning strike covariate but included atmospheric stability covariates as *proxies* for lightning strikes. Note that, OLCF did not include the atmospheric stability covariates. The PLCF and HCF models were trained on data from 1981–2008 (10.69 million observations), leaving the years 2009–2014 for testing the fitted models. The OLCF was trained on data from years 1999–2008 and 2010–2013 (5.12 million observations), leaving the years 2009 and 2014 for testing. Number of cases in the training dataset under HCF, PLCF and OLCF were 22,525; 24,392 and 10,128; respectively. The case-control class-imbalance ratio for the HCF, PLCF and OLCF models were therefore approximately 1:469, 1:433 and 1:504, respectively. Nadeem et al. [29] employed response-based sampling to handle this severe class-imbalance and used *p*_*drop*_ to perform variable selection. We emphasise that our selection methodology in this study is instead based on *Rank*(*X*_*i*_), which works for both Lasso and Ridge regularization.

We illustrate our SVRS algorithm by reanalyzing HCF, PLCF and OLCF logistic regression models using both Lasso and Ridge regularization where we also permute a subset of variables that were deemed unimportant in Nadeem et al. [29]. Permuting a covariate destroys its relationship with the observed response vector and is widely implemented to assess variable importance in regression and classification models, e.g. random forests [47]. Here, barring the 36, 32 and 27 highest ranked covariates in HCF, PLCF, and OLCF models respectively, as reported in Nadeem et al. [29], all the remaining candidate variables are permuted before implementing the SVRS algorithm. Total number of candidate variables included in HCF, PLCF, and OLCF are 82, 69 and 67 respectively (Table 7).

**Table 7:** Case study. Number of permuted and unpermuted covariates, number of permuted covariates classified as important (false positives, *FP*), and number of unpermuted covariates classified as important (*UI*). *FP* and *UI* values outside and inside the parentheses correspond to *λ*_1*se*_ and *λ*_*min*_, respectively. The results are based on *M* = 500 balanced datasets.

Fire Occurrence Model	Number of Covariates	Lasso		Ridge	
	Permuted/Unpermuted	*FP*	*UI*	*FP*	*UI*
HCF	46/36	0 (0)	16 (25)	0 (0)	30 (30)
PLCF	37/32	0 (0)	29 (28)	0 (0)	29 (29)
OLCF	40/27	0 (0)	21 (24)	0 (0)	23 (22)

**Fig 7:**
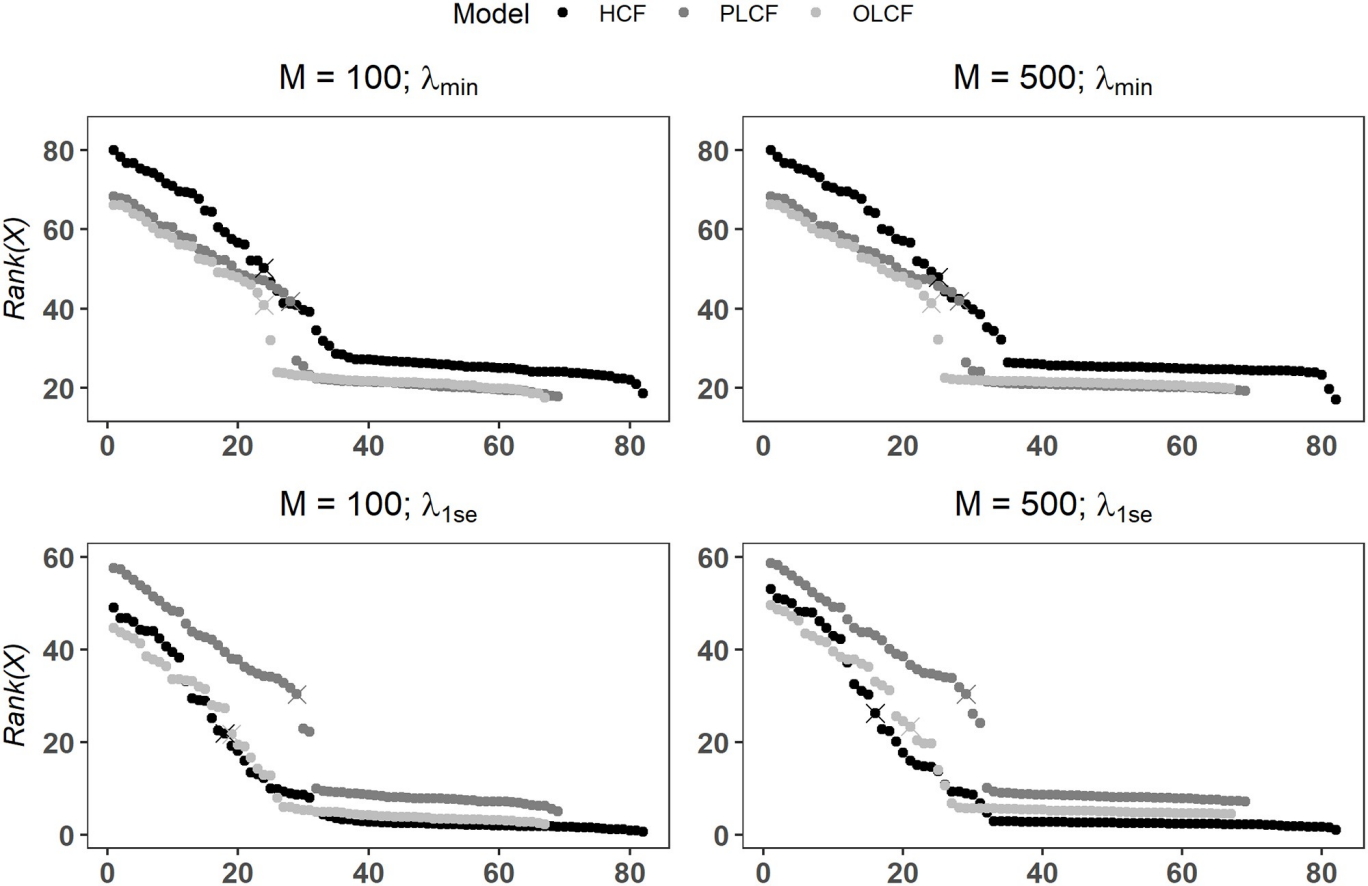
Lasso regularization-based variable rank scores computed using SVRS algorithm for various wildland fire occurrence models. Symbol × marks a changepoint point in sorted *Rank*(*X*) values where variables falling to the right of × are classified be noise covariates.

**Fig 8:**
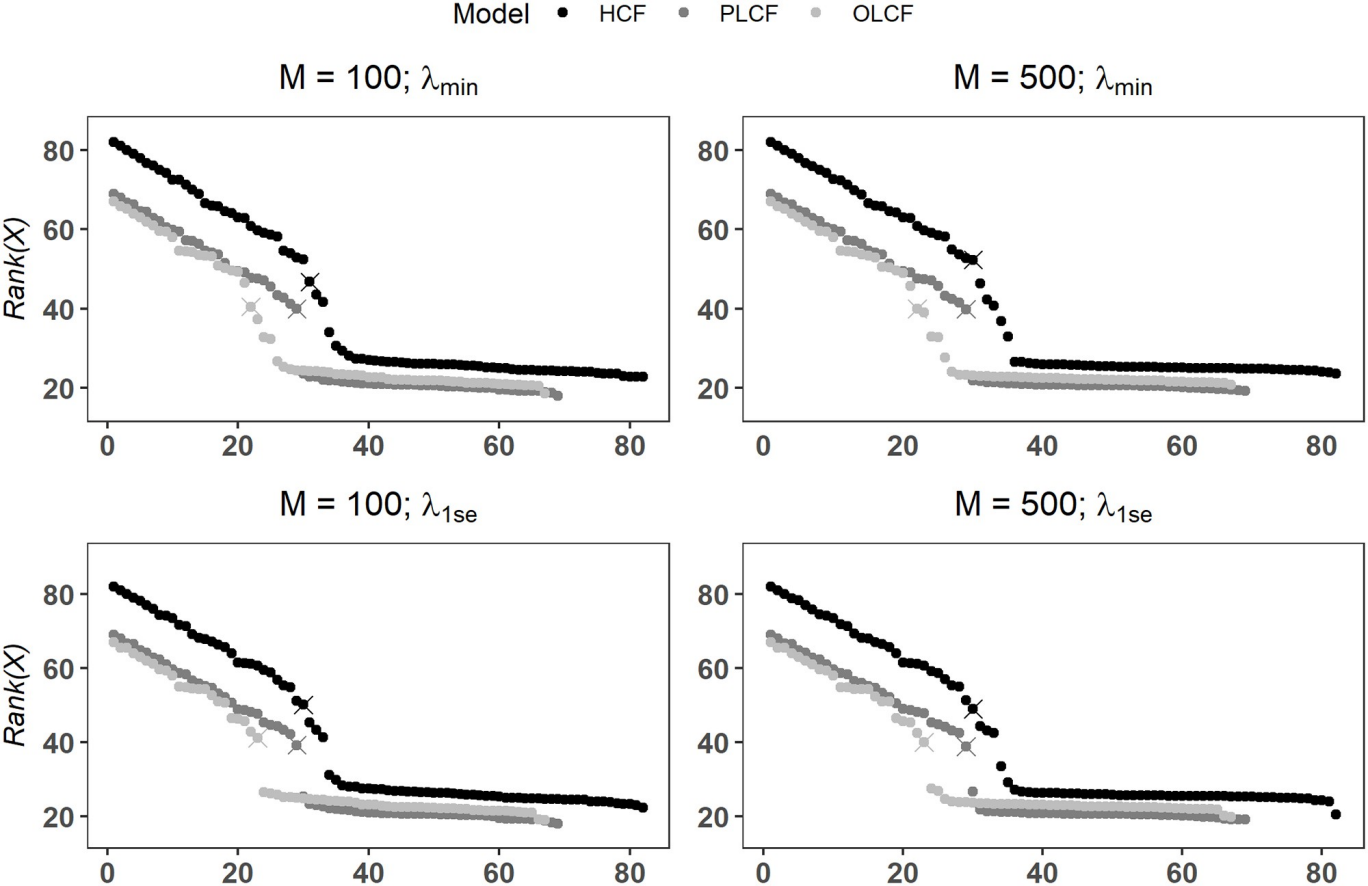
Ridge regularization-based variable ranks scores computed using SVRS algorithm for various wildland fire occurrence models. Symbol × marks a changepoint point in sorted *Rank*(*X*) values where variables falling to the right of × are classified as noise covariates.

Figs 7 and 8 plot *Rank*(*X*_*i*_) scores for the various models along with selection thresholds computed via the changepoint detection method. We also assess change in selection performance with low and high number of downsampled balanced datasets. We further consider the effect of choosing the optimally regularized model as compared to the more conventional practice of choosing a parsimonious model corresponding to tuning parameter (*λ*) value that satisfies the *one-standard-error* rule [48–50]. Following the terminology used in the *glment* R package, we denote the *λ* values corresponding the best and one-standard-error based models as *λ*_*min*_ and *λ*_1*se*_ in Figs 7 and 8 and Table 7.

**Fig 9:**
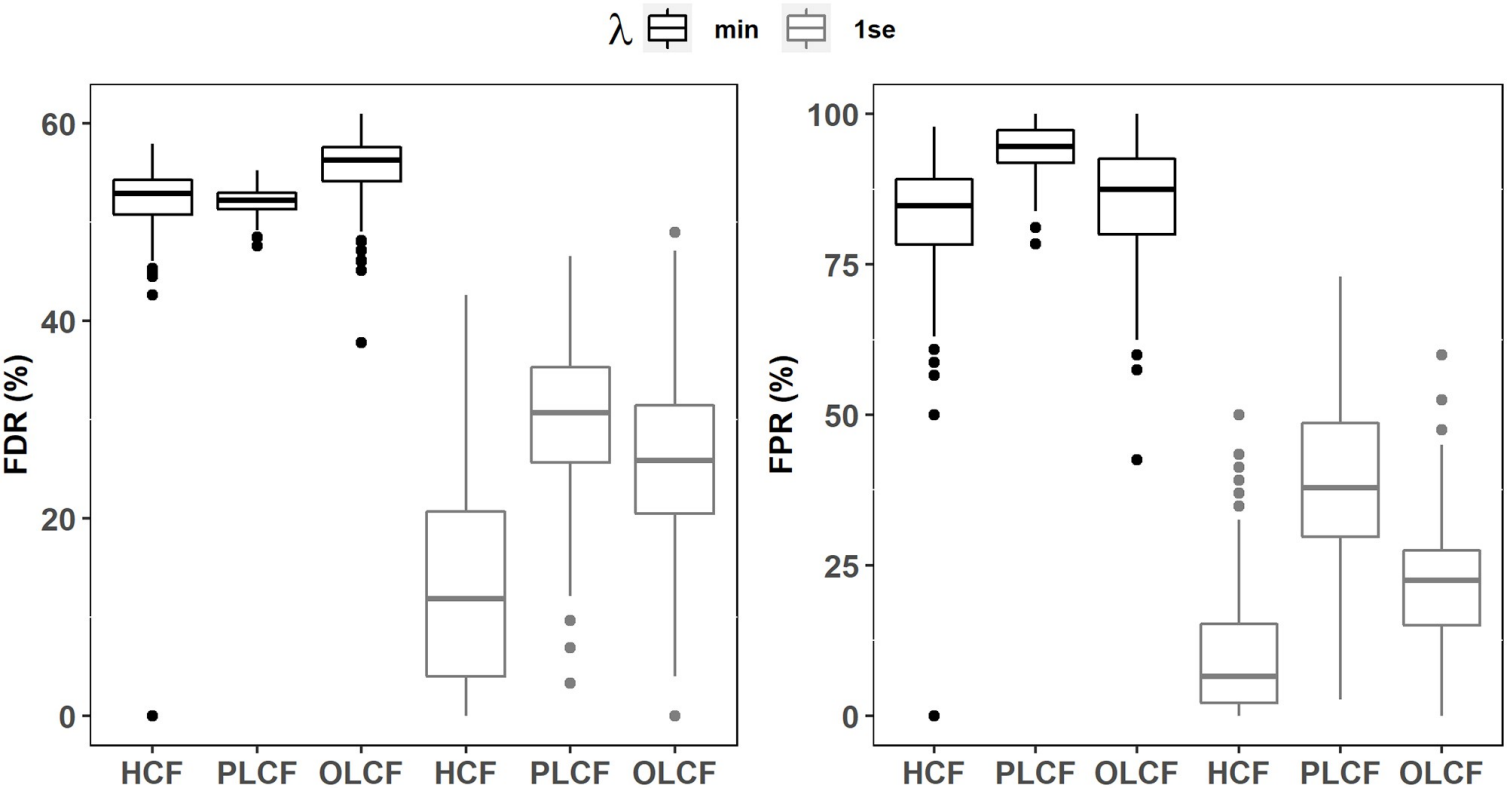
Lasso regularization-based distributions of FDR and FPR for variable selection performed across 500 individual model fits to permuted and balanced wildland fire occurrence datasets.

Regardless of the choice of the tuning parameter and number of balanced datasets (*M*) used, selected threshold values for HCF, PLCF and OLCF models in nearly all cases show that there is a marked change (or a breakpoint) in sorted *Rank*(*X*_*i*_) scores which naturally separates the candidate covariates in two mutually exclusive sets of important (signal) and unimportant (noise) covariates (Figs 7 and 8). It is also evident that the changepoint method accurately detects these breakpoints as shown by the symbols ×. The key finding here is that all permuted covariates are classified as unimportant under Lasso and Ridge regression for *λ*_*min*_ and *λ*_1*se*_ (Table 7; *M* = 500), providing strong evidence that our variable selection approach achieves very low FPR and FDR scores for complex real-world datasets. Notice that this result remains unchanged with *M* = 100 for both Lasso and Ridge regularization methods. On the other hand, FDR and FPR scores computed from the individual Lasso fits are at unacceptably high levels as shown in Fig 9 even when *λ*_1*se*_ is used to force a stronger penalization of the regression coefficients. This finding is inline with the behavior observed in the simulation experiments where individual fit based FDR and FPR values for Lasso/adaLasso were highly elevated under all simulation models. Furthermore, we found that a large proportion of unpermuted covariates are deemed important which agrees with the variable selection results obtained by Nadeem et al. [29] based on Lasso-logistic modeling of the original unperturbed dataset (Table 7). We refer the reader to Nadeem et al. [29] for a detailed discussion of the relevance of selected covariates to the wildland fire occurrence process in British Columbia, Canada.

## Discussion

This study presents a novel variable ranking and selection method, SVRS, for regularized logistic regression in the context of severely imbalanced and potentially massive and high-dimensional datasets. It consists of three basic components: i) a base regularization method, e.g. Ridge regression, that outputs regularized estimates of regression coefficients, ii) response-based sampling to generate an ensemble of regularized coefficients by fitting the base algorithm to several balanced datasets, and iii) an algorithm to stabilize the variable rank scores from the ensemble to select variables having with high rank scores. The method is very flexible as we demonstrate its applicability for regularization methods that enforce both hard- and soft-shrinkage of the regression coefficients. The simulation experiments show that methods with built-in structure estimation, such as Lasso, can produce highly instable and misleading selection results for high-dimensional data. Analysis of permuted wildland fire occurrence data further reveals that these methods can fail spectacularly at controlling the false discovery rate. SVRS, on the other hand, stabilizes the noise in estimated regression coefficients and yields variable selection with high accuracy and very low false discovery rate.

Another potential general regularization framework for logistic regression is the elastic net penalty [6], which includes Lasso and Ridge penalties as the special case. It involves an additional mixing parameter, 0 ≤ *α* ≤ 1; where values of 0 and 1 correspond to Ridge and Lasso penalties, respectively. Here, *α* induces a tradeoff between *l*_2_-norm (Lasso) and *l*_1_-norm (ridge) and works well in the presence of strongly correlated covariates. We studied the performance SVRS algorithm with elastic net in a small simulation experiment under various settings as defined in Tables 1 and 2 and found that the results (not reported here) were similar to Lasso and Ridge cases examined herein. We therefore opted to focus on the two widely applicable special cases of elastic net to simplify the exposition of our methodology.

Use of response-based downsampling in our work is an instance of the general notion of subsampling and is particularly useful in reducing the computational burden when dealing with prohibitively large datasets with extreme class-imbalance. For instance, the full BC wildland fire occurrence dataset for person-caused fires comprises 13 million instances with a case-control class-imbalance ratio of 1:469, whereas a single balanced dataset generated via response-based sampling consists of 45,050 observations only. The methodology introduced herein is in the same vein as stability selection [34, 51], which is a general subsampling based variable selection approach that is designed to improve selection performance of a base selection algorithm for high-dimensional data. However, SVRS differs from stability selection in the following two aspects: i) it stabilizes the regression coefficients across the ensemble whereas the latter is designed to stabilize the selections (i.e. classification of a variables into noise or signal group) performed on subsampled data; and ii) response-based sampling in SVRS ensures that the size of the subsamples can potentially be several hundreds of order of magnitude smaller than [*n*/2], which is the required size to implement stability selection. This latter aspect is crucial in the context of severely imbalanced and massive datasets as training the base algorithm repeatedly on millions of observations can be computationally prohibitive. It is also important to note that response-based sampling approach in SVRS alleviates class-imbalance in individual subsamples without affecting the predictive performance of the base regularization method, whereas the usual random sampling as implemented in stability selection inherits the same severe class-imbalance present in the original dataset.

In summary, this study introduces a new variable selection method for logistic regression modeling of extreme rare events data. The method combines response-based subsampling and commonly employed regularization methods to perform accurate variable selection for high-dimensional and large datasets. The performance results are supported by an extensive simulation experiment and analysis of big and severely imbalanced real-life datasets.

## Supporting information

S1 Appendix Analysis of a reduced case study dataset. (DOCX)
