# Supplementary material for: Stable variable ranking and selection in regularized logistic regression for severely imbalanced big binary data
Source: PLoS One. 2023 Jan 17;18(1):e0280258. doi: 10.1371/journal.pone.0280258 (PMC9844919; doi:10.1371/journal.pone.0280258)
Supplement: S1 Appendix — (DOCX) [file pone.0280258.s001.docx]

**Appendix S1: Analysis of a Reduced Case Study Dataset**

**Article Tittle: Stable variable ranking and selection in regularized logistic regression for severely imbalanced big binary data**

Authors: Khurram Nadeem*, Mehdi-Abderrahman Jabri

University of Guelph, 50 Stone Road E, Guelph, ON, N1G 2W1, Canada.

*Corresponding author.

Email addresses: nadeemk@uoguelph.ca (KN), mehdiabd@uoguelph.ca (MJ).

**1. Case Study: Wildland Fire Occurrence Analysis based on a *Reduced Dataset***

**1.1 Data**

The analysis reported in the main article under section titled “*Case Study: Analysis of Wildland Fire Occurrence Data*” are based on the full wildland fire occurrence dataset (approximately 13 million 1-day space–time voxels spanning 2541 grid cells and 34 fire seasons (1981–2014)). We do not have permission to share this dataset publicly as it is the property of Government of Canada (Natural Resources Canada (NRCan)). However, NRCan has allowed us to share a subset of this data which comprises around 10% (about 1.3 million randomly selected space-time voxels) of the original full dataset.

We provide the following data as part of this supporting information.

**Dataset A1**: The following files are included in this dataset:

*Dataset_A1.csv*: This includes the reduced wildland file data consisting of about 1.3 million observations (10% of the original dataset). Note that “Lightning Caused Occurrence” and “Human Caused Occurrence” are the binary response variables corresponding to the OLCF (and PCLS) and HCF models, respectively. See Table 1 in Nadeem et al. [1] for the description of the remaining variables.

Notice as well that because the reduced dataset is a random sample of drawn from the full wildland fire dataset, the severity of the case-control lass-imbalance ratios persist in the reduced dataset as well. These ratios in the reduced dataset are 1:523, 1:429 and 1:464 for HCF, PLCF and OLCF models, respectively.

**Dataset A2**: This includes raw regression coefficients and the resulting ;SVRS based rank scores under various scenarios. These results were generated by analyzing the data in *Dataset_A1.csv*.

There are two types of result files:

1. *ranks_firex _permuted_regularizedx_Bx_Lx_10pct.csv; and*
2. *betas_firex _permuted_Lasso_B500_Lx_10pct.csv;*

which correspond to rank scores, $Rank\left( X_{i} \right)$, and the regression coefficients obtained by fitting M=500 logistic regression models.

The nomenclature used in naming the files is as follows:

*firex* takes 3 wildland fire occurrences models (HCF, PLCF, OLCF);

*regularizedx* takes 2 regularization methods (lasso, ridge);

*Bx* takes 2 values for the number of model fits (B100 for M=100; and B500 for M=500); and

*Lx* takes 2 values of the regularization parameter $\lambda$ ($\lambda_{1se}, \lambda_{min})$.

Hence, there are 30 files in this data folder, e.g.

*ranks_HCF_permuted_ridge_B500_Lmin_10pct.csv*;

*ranks_PLCF_permuted_lasso_B100_L1se_10pct.csv*;

*betas_OLCF _permuted_Lasso_B500_Lmin_10pct.csv* ; etc.

**Dataset B**: This consists of result files generated by analyzing the *full* *dataset* (the corresponding results appear in the main article). The nomenclature used in naming the files is the same as used for **Dataset A2**.

**1.2 R Code**

*Dataset_A.R*: Code to reproduce the results based on the application of SVRS algorithm on the reduced dataset (*Dataset_A1.csv*). The file is included in **Dataset A2**.

*Dataset_B.R*: Code to reproduce the results based on the application of SVRS algorithm on the *full* dataset. The file is included in **Dataset B**.

**2. Results based on the Reduced Dataset**

Here, we reproduce the wildland fire case study analysis results, as reported in the main article, based on the *reduced* (10%) dataset (*Dataset_A1.csv*). Note that these results can be reproduced by running the code in *Dataset_A.R*.

The following results are included herein:

S1 Table 1: This corresponds to Table 7 in the main article.

S1 Fig 1: This corresponds to Fig 7 in the main article.

S1 Fig 2: This corresponds to Fig 8 in the main article.

S1 Fig 3: This corresponds to Fig 9 in the main article.

**S1 Table 1.**  **Case Study based on the reduced dataset^*^.** Number of permuted and unpermuted covariates, number of permuted covariates classified as important (false positives, $FP$), and number of unpermuted covariates classified as important ($UI$). $FP$ and $UI$ values outside and inside the parentheses correspond to $\lambda_{1se}$ and $\lambda_{min}$, respectively. The results are based on $M=500$ balanced datasets.

| Fire Occurrence Model | Number of Covariates |  | Lasso | |  | Ridge | |
| --- | --- | --- | --- | --- | --- | --- | --- |
|  | Permuted/Unpermuted |  | $FP$ | $UI$ |  | $FP$ | $UI$ |
| HCF | 46/36 |  | 0 (0) | 16 (25) |  | 0 (0) | 30 (30) |
| PLCF | 37/32 |  | 0 (0) | 29 (28) |  | 0 (0) | 29 (29) |
| OLCF | 40/27 |  | 0 (0) | 21 (24) |  | 0 (0) | 23 (22) |

* These results are the same as reported in Table 7. Therefore, as the results remain unchanged, SVRS algorithm remained robust to a substantial reduction in the overall sample size as the reduced dataset is only 10% of the full data analyzed in the main text.


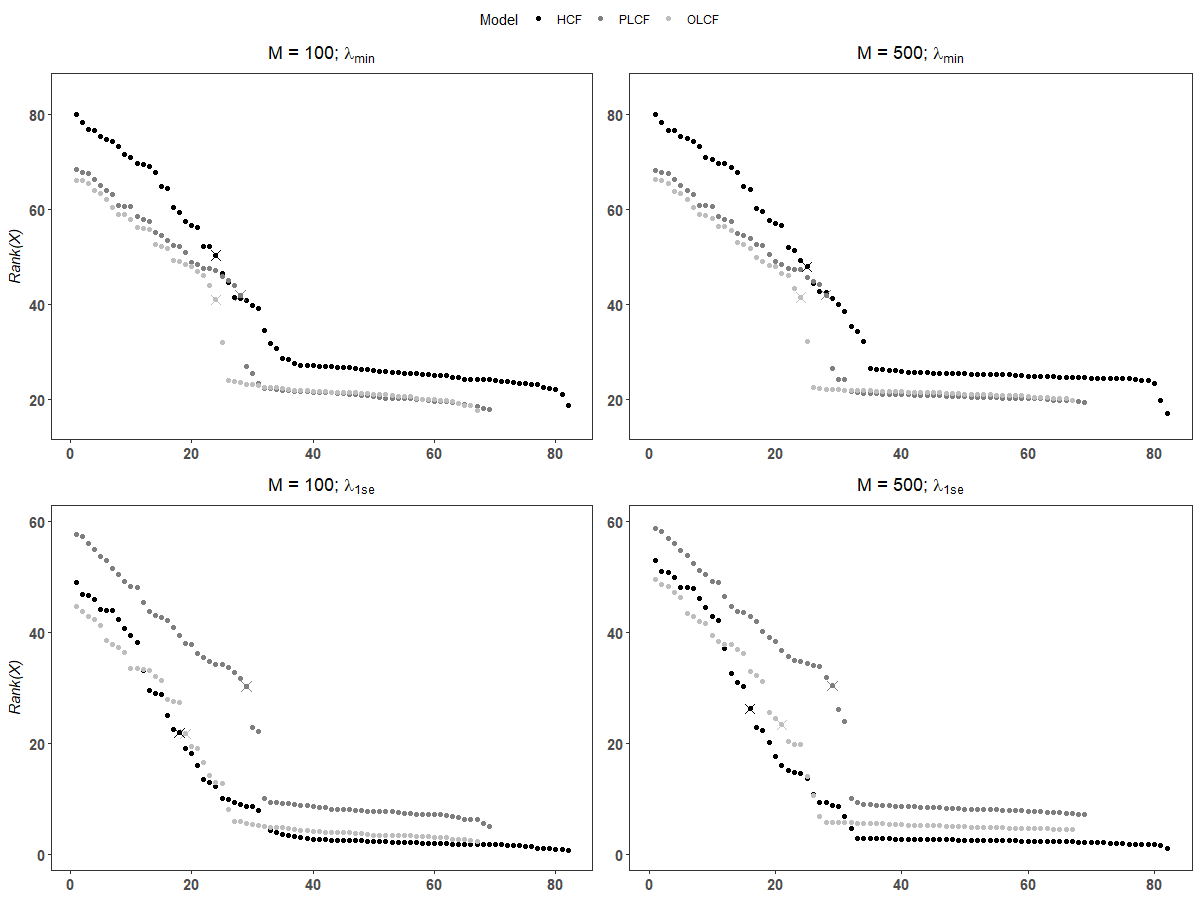


**S1 Fig 1.** **Case Study based on the reduced dataset.** Lasso regularization-based variable rank scores computed using SVRS algorithm for various wildland fire occurrence models. Symbol × marks a changepoint point in sorted Rank(X) values where variables falling to the right of × are classified be noise covariates.


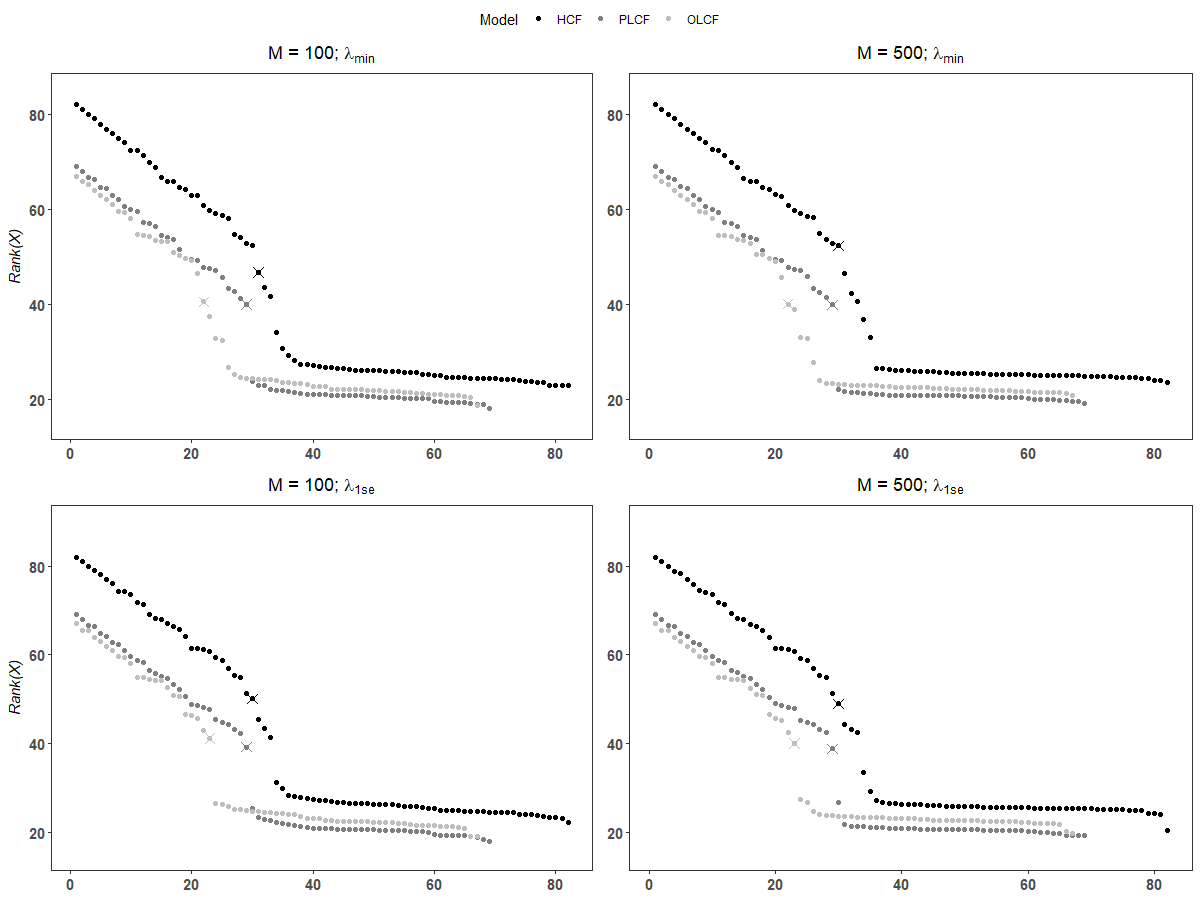


**S1 Fig 2.** **Case Study based on the reduced dataset.** Ridge regularization-based variable ranks scores computed using SVRS algorithm for various wildland fire occurrence models. Symbol × marks a changepoint point in sorted Rank(X) values where variables falling to the right of × are classified as noise covariates.


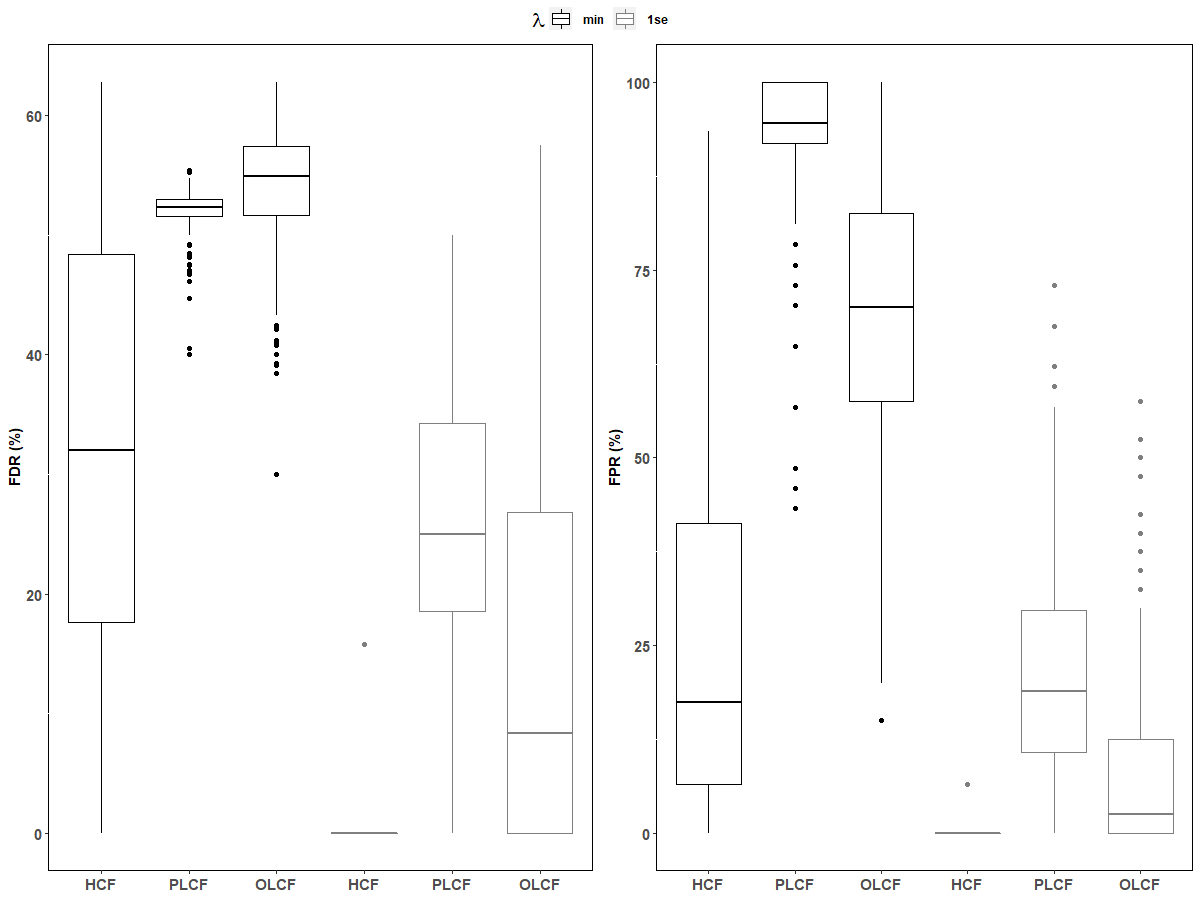


**S1 Fig 3.** **Case Study based on the reduced dataset.** Lasso regularization-based distributions of FDR and FPR for variable selection performed across 500 individual model fits to permuted and balanced wildland fire occurrence datasets.

**Remark**: S1 Fig 3 shows that FDR and FPR scores, as computed from the induvial Lasso model fits, are zero in almost all of the 500 model fits. This in contrast to the *full data* results depicted in Fig 9, where the median values of these scores are much higher with large variability. This provides further evidence that that the variable selection based on the individual Lasso logistic regression fits can be highly sensitive to sampling variability (recall that S1 Fig 3 results based on a 10% random sample of the full dataset). In contrast, our SVRS results remain unchanged as shown in Table 7 and S1-Table 1.

Furthermore, for the individual model fits, the average proportion of *unpermuted* HCF covariates (under $\lambda_{min}$) classified as unimportant is 80% (the proportion defined as the fraction, $1-\frac{UI}{36}$ , in terms of S1-Table 1 notation); whereas the corresponding proportion under SVRS algorithm is only 31% (S1-Table 1). This shows that the individual HCF Lasso models lack power in detecting important covariates when trained with a much smaller/reduced sample size. This in turn explains why HCF has very small FDR and FPR scores in S1 Fig 3.

**References**

1. Nadeem K, Taylor SW, Woolford DG, Dean CB. Mesoscale spatiotemporal predictive models of daily human-and lightning-caused wildland fire occurrence in British Columbia. International journal of wildland fire. 2019 Dec 24;29(1):11-27. https://doi.org/10.1071/WF19058
